# Influence of La Doping on the Magnetic Properties of the Two-Dimensional Spin-Gapped System SrCu_2_(BO_3_)_2_

**DOI:** 10.1021/acs.inorgchem.5c04249

**Published:** 2025-12-29

**Authors:** Lia Šibav, Tilen Knaflič, Graham King, Zvonko Jagličić, Maja Koblar, Kirill Povarov, Sergei Zvyagin, Denis Arčon, Mirela Dragomir

**Affiliations:** † 61790 Jožef Stefan Institute, Jamova Cesta 39, Ljubljana 1000, Slovenia; ‡ Jožef Stefan International Postgraduate School, Jamova Cesta 39, Ljubljana 1000, Slovenia; § 117197 Canadian Light Source, 44 Innovation Blvd, Saskatoon, SK S7N 2 V3, Canada; ∥ Institute of Mathematics, Physics and Mechanics, Jadranska Ulica 19, 1000 Ljubljana, Slovenia; ⊥ Faculty of Civil and Geodetic Engineering, University of Ljubljana, Jamova Cesta 2, Ljubljana 1000, Slovenia; # Dresden High Magnetic Field Laboratory (HLD-EMFL) and Würzburg-Dresden Cluster of Excellence ct.Qmat, Helmholtzzentrum Dresden-Rossendorf, Dresden 01328, Germany; ∇ Faculty of Mathematics and Physics, University of Ljubljana, Jadranska Ulica 19, Ljubljana 1000, Slovenia

## Abstract

Doping of the two-dimensional dimer antiferromagnet SrCu_2_(BO_3_)_2_ has long been proposed as a potential route toward realizing resonating valence bond superconductivity in this system; however, experimental progress has remained limited. This study explores the effects of La doping on the ground state of SrCu_2_(BO_3_)_2_ and reports the first flux growth of Sr_1–*x*_La_*x*_Cu_2_(BO_3_)_2_ single crystals with nominal doping levels up to *x* = 0.15. Powder X-ray diffraction and energy-dispersive X-ray spectroscopy confirm the successful incorporation of La on the Sr sites within the same tetragonal *I* 4̅ 2*m* structure, although the effective doping was found to be <50% of the nominal concentration. La doping induces systematic changes in the magnetic properties of SrCu_2_(BO_3_)_2_, with a reduction of the effective spin gap from 28.2 K for the undoped sample to 20.3 K for *x* = 0.15, as determined from the low-temperature magnetic susceptibility. The X-band electron spin resonance measurements reveal the emergence of unpaired Cu^2+^ spins, which develop antiferromagnetic correlations below ∼5.5 K. These findings corroborate the breaking of the local spin dimers in SrCu_2_(BO_3_)_2_ induced by La doping. No superconductivity is observed across the entire doping range studied.

## Graphical abstract

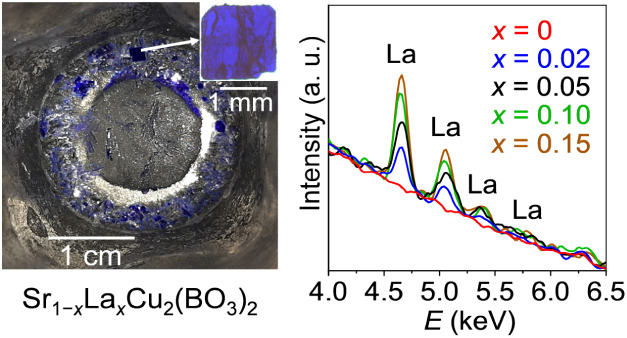

## 1 Introduction

In the last decades, considerable research has been devoted to low-dimensional quantum magnets, in particular, to those exhibiting a spin gap in their excitation spectra. In this context, charge-transfer insulators, such as Cu^2+^ oxides, have been extensively investigated due to their connection to cuprate superconductors. The discovery of high-*T*_C_ superconductivity in charge-doped cuprates in 1986 ^1^ indicated the potential of chemical doping as an avenue for accessing novel exotic ground states in low-dimensional and frustrated quantum magnets.^2^ This milestone spurred extensive theoretical work, proposing mechanisms for unconventional superconductivity, such as the resonating valence bond (RVB) model on the 2D frustrated spin lattice,^3,4^ which predicts that upon doping, the charge-transfer gap closes. This would allow previously localized spin-singlet pairs (analogous to Cooper pairs) to propagate freely, leading to the onset of superconductivity.

While there have been several studies on gapped one-dimensional (1D) magnetic systems with spin-singlet ground states, such as spin-Peierls,^5^ Haldane chains,^6^ or spin ladders,^7^ there are less examples of two-dimensional (2D) spin-gapped systems.^8–10^ Thus, systems with a 2D spin-singlet ground state and frustrated spin configuration are of great interest. One notable representative of such systems is the frustrated antiferromagnet SrCu_2_(BO_3_)_2_.^11–13^ The unique structure of this material consists of Cu^2+^ ions (*S* = 1/2), which form a two-dimensional orthogonal dimer lattice that stacks along the *c*-axis. Each Cu^2+^–Cu^2+^ pair constitutes a strongly antiferromagnetically coupled dimer within the *ab* plane, with the next-nearest exchange to the neighboring dimers comparable in strength. The resulting network maps directly onto the Shastry–Sutherland model, describing a frustrated quantum spin system characterized by competing intradimer (*J*) and interdimer (*J*′) antiferromagnetic exchange interactions.^14–16^ Due to the orthogonal arrangement of adjacent dimers, when the ratio *J*′/*J* is sufficiently small, i.e., *J’*/*J* < 0.69, the model takes an exact ground state comprising a product of singlet states on each dimer.^13^ For intermediate values 0.86 > *J’*/*J* > 0.69, a gapped plaquette singlet phase emerges.^17^ For *J*′/*J* > 0.86, the system transitions to a Néel antiferromagnetic state,^17^ with a quantum spin liquid phase possibly stabilized between the gapped plaquette-singlet and magnetically ordered regimes.^18,19^

Experimental values for *J’*/*J =* 0.68 ^17^ locate SrCu_2_(BO_3_)_2_ very close to the quantum phase boundaries either to a Néel ordered state or to the plaquette singlet state. Various experiments provide firm evidence for a dimer spin-singlet ground state with the gap Δ = 34(1) K.^20–22^ Magnetization curves collected at temperatures well below the gap show quantized plateaus at 1/3, 1/4, or 1/8 of the Cu saturation moment due to the localized nature of excited triplets.^12,23^ The origin of these plateaus was attributed to the nearly localized nature of the triplet excitations.^13^

Both external pressure and chemical doping have proven effective in tuning the magnetic ground state of SrCu_2_(BO_3_)_2_; pressure reduces the spin gap by modifying exchange interactions and pushing the system closer to phase boundaries,^24–29^ while magnetic dilution—such as Mg substitution for Cu—suppresses the spin gap by disrupting the singlet dimer order.^30^ According to theoretical predictions, a resonating valence bond superconductor might be realized as the ground state of SrCu_2_(BO_3_)_2_ upon A-site electron or hole doping—specifically, by substituting the interlayer Sr^2+^ with an aliovalent ion, M^3+^ or M^+^, respectively, resulting in Sr_1−*x*_M_*x*_Cu_2_(BO_3_)_2_.^31–33^ However, despite its potential, A-site chemical substitution in SrCu_2_(BO_3_)_2_ remains remarkably difficult. Previous studies on polycrystalline samples with various dopants, i.e., Ca, Ba, Al, La, Na, or Y, showed only subtle structural and magnetic changes, as the effective doping concentrations are consistently low. Moreover, challenges with efficient dopant incorporation and impurity phases highlighted the need for single crystals.^34,35^ The optical floating zone method enabled the growth of doped single crystals with dopants such as Ba, Na, or La,^36–38^ but these efforts echoed similar challenges: crystal growth was slow, highly sensitive to chemical composition and growth conditions, and often resulted in multigrain samples. Each dopant significantly altered the growth parameters, making reproducible, uniform doping difficult, even at low dopant concentrations. Similar issues were reported for B-site doping with isovalent, nonmagnetic ions,^30,39–41^ emphasizing the need for further research and alternative growth methods.

Motivated by reports that La induces the most significant reduction of the spin gap among the A-site dopants,^35^ this work focuses on La doping of SrCu_2_(BO_3_)_2_. Previous investigations of both polycrystalline and single-crystalline samples—such as those prepared by Liu et al.^35^ and Dabkowska et al.,^37^ respectively—were limited in doping range, reaching only up to nominal *x* = 0.10, and hindered by complications including secondary phase formation and instabilities of the crystal growth. In this work, a flux method, previously used for the growth of submillimeter single crystals of undoped SrCu_2_(BO_3_)_2_ using LiBO_2_ as a flux,^11^ was optimized to grow plate-like Sr_1–*x*_La_*x*_Cu_2_(BO_3_)_2_ single crystals (with *x* = 0.02, 0.03, 0.04, 0.05, 0.10, and 0.15), reaching lateral sizes up to 3 mm. La incorporation into the parent structure was unambiguously confirmed by powder X-ray diffraction (PXRD) and energy-dispersive X-ray spectroscopy (EDS). It was found that the effective doping was <50% of the nominal value. The limited incorporation of La nonetheless induced progressive changes in magnetic behavior. A gradual reduction of the effective spin gap—from 28.2 K for undoped SrCu_2_(BO_3_)_2_ to 20.3 K at *x* = 0.15—was observed, accompanied by the emergence of dimer-free Cu^2+^ spins at low temperatures. X-band and high-field electron spin resonance (ESR) spectroscopy further showed evidence of antiferromagnetic exchange interactions between these spins below 5.5 K, which primarily account for the observed effective spin-gap reduction at low doping levels, while the intrinsic dimer lattice spin dynamics remain largely preserved.

## 2 Experimental Section

### 2.1 Synthesis

#### 2.1.1 Solid-State Synthesis

Polycrystalline Sr_1–*x*_La_*x*_Cu_2_(BO_3_)_2_ samples with nominal *x* concentrations of 0, 0.01, 0.02, 0.03, 0.04, 0.05, 0.10, and 0.15 were first prepared using a conventional solid-state method.^30^ High-purity SrCO_3_ (Alfa Aesar, 99.994%), CuO (Aldrich, 99.99%), H_3_BO_3_ (Alfa Aesar, 99.9995%), and La_2_O_3_ (Alfa Division, 99.99%) were used as starting materials. Prior to use, La_2_O_3_ was preannealed in air at 1000 °C for 48 h.

The corresponding reactions can be described by the following equations:

1 $${\mathrm{SrCO}}_{3}+2\mathrm{CuO}+2H_{3}{\mathrm{BO}}_{3}\to {\mathrm{SrCu}}_{2}{\left({\mathrm{BO}}_{3}\right)}_{2}+{\mathrm{CO}}_{2}+3H_{2}O$$

2 $$1-x{\mathrm{SrCO}}_{3}+2\mathrm{CuO}+2H_{3}{\mathrm{BO}}_{3}+x/{\mathrm{2 La}}_{2}O_{3}\to {\mathrm{Sr}}_{1-x}{\mathrm{La}}_{x}{\mathrm{Cu}}_{2}{\left({\mathrm{BO}}_{3}\right)}_{2}+1-x{\mathrm{CO}}_{2}+3H_{2}O$$

#### 2.1.2 Single-Crystal Growth

Single-crystal growth experiments were performed by optimizing a previously reported flux method^11^ for growing undoped SrCu_2_(BO_3_)_2_ for structure solution using lithium metaborate, LiBO_2_, as the flux. Here, blue Sr_1–*x*_La_*x*_Cu_2_(BO_3_)_2_ powders of nominal doping concentrations *x* = 0, 0.02, 0.03, 0.04, 0.05, 0.10, and 0.15 that resulted from solid-state reactions were used as starting materials.

In a typical flux growth experiment, between 0.25 and 0.50 g of polycrystalline Sr_1–*x*_La_*x*_Cu_2_(BO_3_)_2_ was hand-homogenized together with the flux in an agate mortar at material-to-flux mass ratios of 2:1, 3:1, 4:1, 5:1, 7:1, or 10:1. The homogenized mixture was transferred into 20 or 30 mL platinum crucibles, covered with a platinum lid, and positioned inside an alumina crucible covered with an alumina cap. The crucible was then heated to 875 °C in a muffle furnace at a heating rate of 100 °C/h and left to dwell for 1, 2, or 3 h, followed by slow cooling to 600 °C with cooling rates of 1, 2, 3, 5, or 10 °C/h before being air-quenched to room temperature. A schematic representation of the experimental setup and the growth process is shown below (Figure 1). The resulting blue thin plate-like single crystals with lateral sizes up to 3 mm were separated from the crucible using deionized water in an ultrasonic bath.

**Figure 1:**
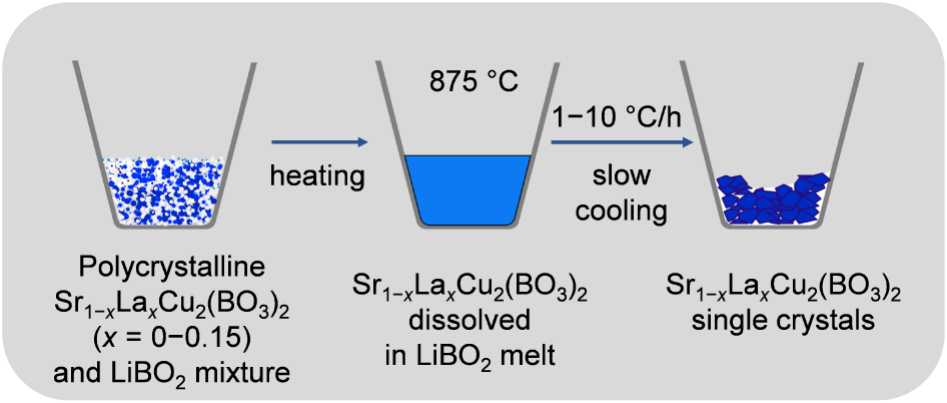
Schematic representation of the experimental setup and process for the Sr_1–*x*_La_*x*_Cu_2_(BO_3_)_2_ flux growth, illustrating three key steps: (left) preparation of the initial polycrystalline mixture of Sr_1–x_La_*x*_Cu_2_(BO_3_)_2_ and LiBO_2_ flux, (middle) dissolution of the mixture in molten flux at a dwell temperature of 875 °C, and (right) formation of Sr_1–*x*_La_*x*_Cu_2_(BO_3_)_2_ single crystals during slow cooling.

### 2.2 Characterization

#### 2.2.1 Powder X-ray Diffraction (PXRD)

The phase composition of undoped and La-doped polycrystalline samples obtained after the solid-state synthesis, as well as powders from crushed single crystals, was first probed using laboratory powder X-ray diffraction (PXRD) with a Panalytical X’Pert Pro powder diffractometer and Cu–Kα1 radiation in the 20–120° 2θ range with a step size of 0.016° and a counting time of 300 s per step. Crushed single crystals were also analyzed by synchrotron PXRD; the data were collected at the Brockhouse high-energy wiggler beamline^42^ at the Canadian Light Source (CLS) using an area detector and λ = 0.3502 Å radiation with Ni as a calibrant. Single crystals of each nominal doping concentration were crushed, placed into Kapton capillaries, measured in a 2θ range of 1–26° and binned into 0.01° steps.

A Rietveld least-squares method was used for structural refinements, which were performed using the program GSAS-II.^43^

#### 2.2.2 Scanning Electron Microscopy (SEM) and Energy-Dispersive X-ray Spectroscopy (EDS)

For chemical analysis and microstructural investigation, Sr_1–*x*_La_*x*_Cu_2_(BO_3_)_2_ single crystals were mounted on carbon tape and carbon coated by using a Balzers SCD 050 sputter coater Balzers SCD 050. The SEM imaging and EDS compositional analyses of the crystals were performed on two instruments: a Thermo Fisher Quanta 650 ESEM equipped with an energy-dispersive X-ray spectrometer (Oxford Instruments, AZtec Live, Ultim Max SDD 65 mm^2^) and a field-emission-gun scanning electron microscope (FE-SEM; JEOL JSM-7600) equipped with an energy-dispersive X-ray spectrometer (EDS; INCA Oxford 350 EDS SDD) and electron backscatter diffraction (EBSD). The accelerating voltage used was 20 kV in all cases.

#### 2.2.3 Magnetic Susceptibility

Magnetic susceptibility measurements were performed on a Quantum Design MPMS3 (undoped SrCu_2_(BO_3_)_2_) and MPMS-XL-5 (La-doped SrCu_2_(BO_3_)_2_) SQUID magnetometers in a temperature interval 2–300 K in a static magnetic field of 1 kG. An undoped SrCu_2_(BO_3_)_2_ single crystal was fixed to a quartz holder with Apiezon-N grease and inserted into the magnetometer. The La-doped SrCu_2_(BO_3_)_2_ single crystals were measured as polycrystalline samples, randomly stacked at the bottom of a Wilmad 4 mm Suprasil ESR sample tube. The temperatures corresponding to the susceptibility maxima, *T*_max_, were determined by fitting the susceptibility data near the peak to a Lorentzian function.

#### 2.2.4 Electron Spin Resonance Spectroscopy (ESR)

Continuous wave (CW) electron spin resonance spectroscopy experiments were performed by using a conventional Bruker E500 spectrometer, operating in the X-band at the resonant frequency ν_L_ of 9.37 GHz. The spectrometer was equipped with a Varian TEM104 dual cavity resonator, an Oxford Instruments ESR900 cryostat, and an Oxford Instruments ITC503 temperature controller.

The measurements were conducted from room temperature down to 4 K with a typical modulation amplitude of 5 G, a modulation frequency between 50 and 100 kHz, and microwave power of 1–2 mW.

For each measurement, approximately 30 mg of polycrystalline undoped SrCu_2_(BO_3_)_2_ and 5–30 mg of La-doped small single crystals (measured as quasi-polycrystalline samples) were placed in 4 mm Suprasil quartz ESR tubes (Wilmad). For samples with *x* = 0.10 and *x* = 0.15, the ESR spectrometer was equipped with a Bruker 4122SHQE cylindrical resonator operating at 9.4 GHz. Due to the higher sensitivity of this resonator, the latter two samples were measured using a low microwave power of 0.1 mW.

High-field ESR measurements were performed at the High Magnetic Field Laboratory (HLD), Helmholtz-Zentrum Dresden Rossendorf (HZDR) employing a transmission-type ESR spectrometer (similar to that described in ref^44^) in magnetic fields up to 16 T. Multiple crystals with a nominal La concentration of 0.03 were stacked using vacuum grease. A set of VDI microwave sources was used, allowing probe of magnetic excitations at different frequencies in the range from 140 to 495 GHz. Prior to the measurements of ESR spectra, the sample was slowly cooled to the base temperature *T* = 1.7–2.9 K.

## 3 Results and Discussion

### 3.1 Synthesis and Crystal Growth

Solid-state synthesis of Sr_1–*x*_La_*x*_Cu_2_(BO_3_)_2_ with *x* = 0.01, 0.02, 0.03, 0.04, 0.05, 0.10, and 0.15 predominantly results in the tetragonal SrCu_2_(BO_3_)_2_ phase with the *I*4̅2*m* space group, as confirmed by laboratory PXRD (Figure 2). A small amount of CuO is also present, consistent with previous reports on undoped polycrystalline samples.^11,45^ A secondary La phase, LaBO_3_, becomes noticeable when *x* ≥ 0.01, suggesting that La does not incorporate into the parent structure but instead forms a La-rich impurity phase. A systematic increase in the concentrations of both LaBO_3_ and CuO with increasing La content is also observed, supporting this conclusion. This is additionally substantiated by the absence of notable peak shifts in the PXRD patterns relative to the undoped sample.

**Figure 2:**
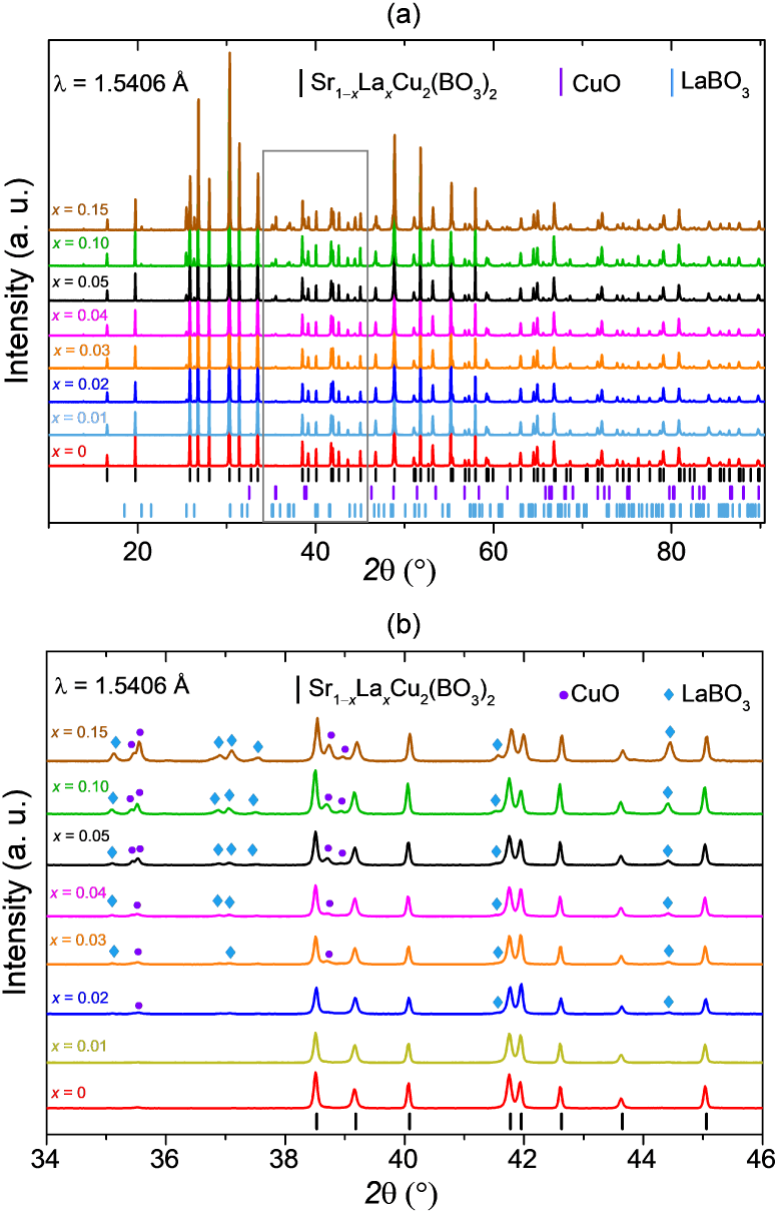
(**a**) Laboratory PXRD patterns of polycrystalline Sr_1–*x*_La_*x*_Cu_2_(BO_3_)_2_ with *x* = 0, 0.01, 0.02, 0.03, 0.04, 0.05, 0.10, and 0.15 as synthesized from the thermal solid-state chemistry route. All doped samples exhibit the main SrCu_2_(BO_3_)_2_ phase, accompanied by minor amounts of unreacted CuO and LaBO_3_ as secondary phases. (**b**) Enlarged view of the 34°−46° 2θ range marked with a gray rectangle in a), highlighting the reflections corresponding to the CuO and LaBO_3_ impurities for improved clarity.

Since a direct solid-state synthesis proved ineffective for incorporating La, single-crystal growth was pursued as an alternative. Given the reported challenges associated with the optical floating zone growth,^37^ the flux method emerged as a more practical and accessible approach for growing these crystals. This technique does not require highly specialized equipment and was considered likely to succeed as it had previously been used to grow crystals of the undoped phase.^11,46,47^ In a previous attempt to synthesize SrCu_2_(BO_3_)_2_ ^11^ crystals intended for structure determination via single-crystal X-ray diffraction (SCXRD), LiBO_2_ was used as flux. Two other subsequent studies reported the use of another borate flux, namely Na_2_B_4_O_7_.^46,47^ In the present study, both fluxes were tested in initial experiments; however, only LiBO_2_ yielded crystals in our setup and was thus chosen for optimization and subsequent growth experiments. The starting materials for these crystal growth experiments were Sr_1–*x*_La_*x*_Cu_2_(BO_3_)_2_ powders prepared via solid-state synthesis.

The material-to-flux mass ratio, dwell time, dwell temperature, and cooling rate were optimized to result in blue plate-like undoped and La-doped SrCu_2_(BO_3_)_2_ single crystals with lateral sizes of up to 3 mm (Figure 3 and Supporting Information, Figure S1). The optimal conditions within the tested parameters (Section 2.2), which yielded the largest crystals, are a material-to-flux mass ratio of 3:1 or 4:1, a dwell time of 2 h at 875 °C, and cooling from 875 to 600 °C at a rate of 2 °C/h, followed by air quenching to room temperature. Material-to-flux mass ratios of 5:1 or higher, as well as 2:1 or lower, result in crystals with minimal sizes. A faster cooling rate of 10 °C/h does not yield single crystals, whereas slower cooling rates of 5 or 3 °C/h produced crystals, albeit smaller compared to those formed at the optimal 2 °C/h rate. Further reduction of the cooling rate to 1 °C/h does not offer any additional benefit in crystal size. In contrast to the dwell time, which shows no notable effect, the material-to-flux mass ratio and cooling rate are identified as key factors influencing the crystal growth. We note that for undoped SrCu_2_(BO_3_)_2_, the resulting crystals are significantly larger than those reported in ref^11^, corroborating a successful optimization of growth conditions in this study.

**Figure 3:**
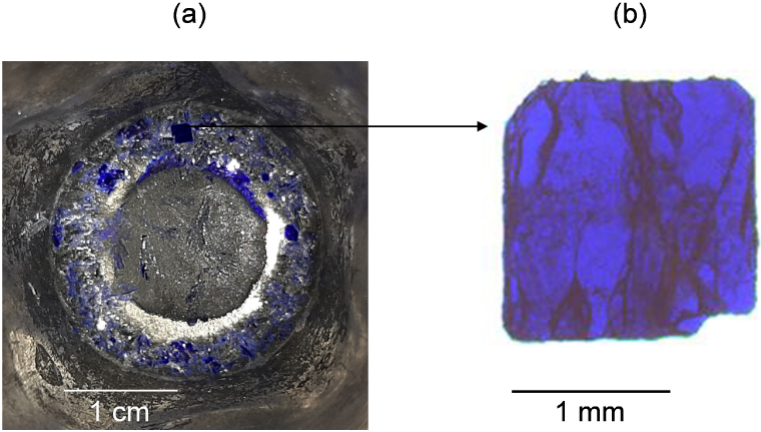
(**a**) Representative image of as-grown Sr_1–*x*_La_*x*_Cu_2_(BO_3_)_2_ single crystals on the walls of a Pt crucible immediately after air quenching. (**b**) Optical image of a selected single crystal after its separation from the flux.

The incorporation of La in the parent SrCu_2_(BO_3_)_2_ structure during single-crystal growth is assessed by PXRD performed on crushed single crystals of Sr_1–*x*_La_*x*_Cu_2_(BO_3_)_2_ crystals with nominal *x* = 0–0.15 (Figure 4a). The absence of La-based secondary phases in normalized synchrotron PXRD patterns indicates a successful incorporation of lanthanum in the parent SrCu_2_(BO_3_)_2_ structure upon single-crystal growth. At the highest nominal concentrations, *x* = 0.10 and 0.15, LaBO_3_ reappears as a minor secondary phase, as previously detected in polycrystalline samples prepared by the thermal solid-state chemistry route. Its presence is likely due to residual unincorporated LaBO_3_ particles remaining on the crystal surfaces, as ultrasonication—used to separate the crystals from the flux after growth—was kept brief to avoid damaging the thin, plate-like crystals. This interpretation is further supported by EDS analysis (discussed in the next section), where LaBO_3_ particles are observed on the surface of crystals with these nominal doping concentrations. Unreacted CuO is detected in all samples, originating from the crystal surfaces, as suggested by EDS analysis and prior reports.^11^

**Figure 4:**
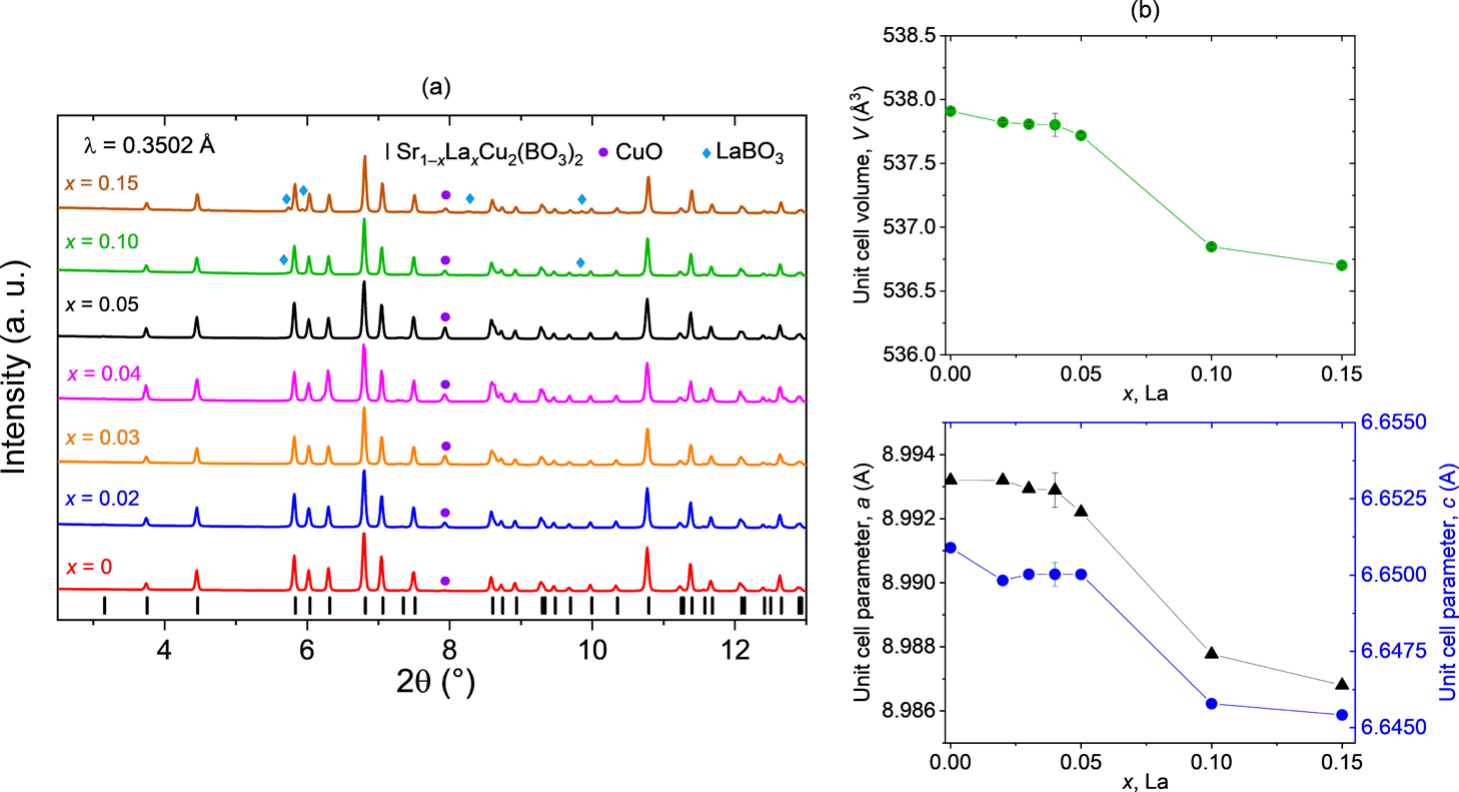
(**a**) Synchrotron (λ = 0.3502 Å) PXRD of Sr_1–*x*_La_*x*_Cu_2_(BO_3_)_2_ crushed single crystals with nominal *x* = 0–0.15. The presence of the main phase, SrCu_2_(BO_3_)_2_, is observed along with a small fraction of unreacted CuO. A few peaks corresponding to the LaBO_3_ secondary phase are also noticed at the highest two nominal concentrations, *x* = 0.10 and *x* = 0.15, with the former being barely detectable. (**b**) The unit cell volume decreases with increasing nominal La doping, consistent with the smaller ionic radius of La^3+^ compared to that of Sr^2+^.

A detailed summary of Rietveld refinement profiles and resulting structural parameters is available in the Supporting Information (Supporting Information, Figure S2 and Tables S1 and S2). Rietveld refinement analyses with the *I*4̅2*m* space group (Supporting Information, Table S1) reveal a decreasing trend in the unit cell parameters and volume as a function of nominal doping concentration (Figure 4b). This trend is consistent with expectations based on the ionic radii of the substituting ions, where Sr^2+^ (1.26 Å, coordination number CN = 8) is partially replaced by La^3+^ (1.16 Å, CN = 8). Specifically, the unit cell volume decreases from 537.91(2) Å^3^ for the undoped sample to 536.70(1) Å^3^ for the sample with nominal *x* = 0.15, corresponding to a slight volume reduction of 0.22%, which suggests minimal but clearly detectable structural distortion. A small decrease in the unit cell volume is observed up to *x* = 0.05, followed by a more pronounced reduction for *x* = 10 and *x* = 0.15. This aligns well with the trends observed in the EDS and magnetic susceptibility measurements, as well as with literature reports on doped crystals with nominal *x* = 0.05, grown by the optical floating zone method,^37^ which also showed a decrease in unit cell volume relative to both undoped ceramics and single crystals.

### 3.2 Scanning Electron Microscopy (SEM) and Energy-Dispersive X-ray Spectroscopy (EDS)

Previous studies on polycrystalline samples indicated that La does not incorporate in the structure during the solid-state synthesis.^48^ This is also confirmed by our findings—see Section 3.1. However, SEM-EDS analyses performed on single crystals unambiguously prove the successful incorporation of La into the parent structure of SrCu_2_(BO_3_)_2_ during single-crystal growth as La is consistently detected for all nominal doping concentrations (Figure 5).

**Figure 5:**
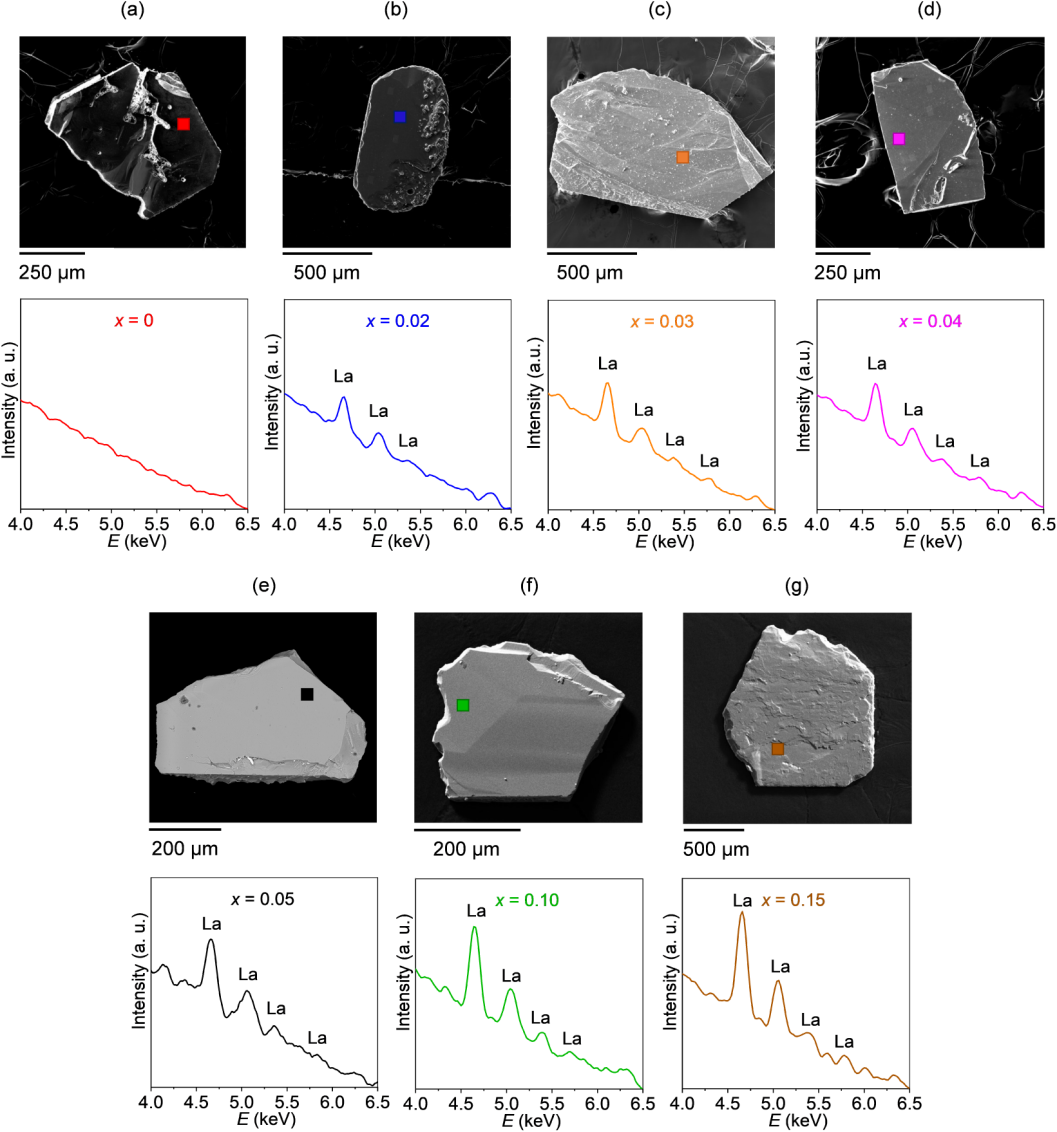
(**a**–**g**) Representative EDS point analysis spectra for Sr_1–*x*_La_*x*_Cu_2_(BO_3_)_2_ single crystals with *x* = 0–0.15. The corresponding SEM images for each doping concentration are displayed above the EDS spectra with squares indicating the selected regions for the point analysis.

The characteristic La emission peaks are clearly observed at 4.65 (Lα) and 5.04 keV (Lβ_1_) in all doped samples. Although two other La peaks, the Lβ_2_ emission at 5.38 keV and the La emission at 5.89 keV, are weaker than the main Lα emission line, they are still detectable for nominal *x* ≥ 0.03. Additionally, a systematic increase in La peak intensity is observed with *x*, peaking at *x* = 0.15. A similar trend is also observed in elemental mapping analyses, which further reveals a relatively homogeneous distribution of La in all doped crystals and a systematic increase in effective doping concentration with nominal *x*.

A representative SEM image, elemental maps, and the summed EDS spectrum for a doped crystal with nominal *x* = 0.05 are shown below (Figure 6), with corresponding data for nominal *x* = 0.04 and 0.10 available in Supporting Information, Figure S3. Secondary phases detected in PXRD—LaBO_3_ and CuO—are also observed on the surfaces of crystals starting from the nominal concentration of *x* = 0.04 for CuO (Supporting Information, Figure S3a) and *x* = 0.10 for LaBO_3_. The LaBO_3_ secondary phase becomes more visible for the highest nominal doping concentration of *x* = 0.15 (Supporting Information, Figure S4), in agreement with the PXRD results.

**Figure 6:**
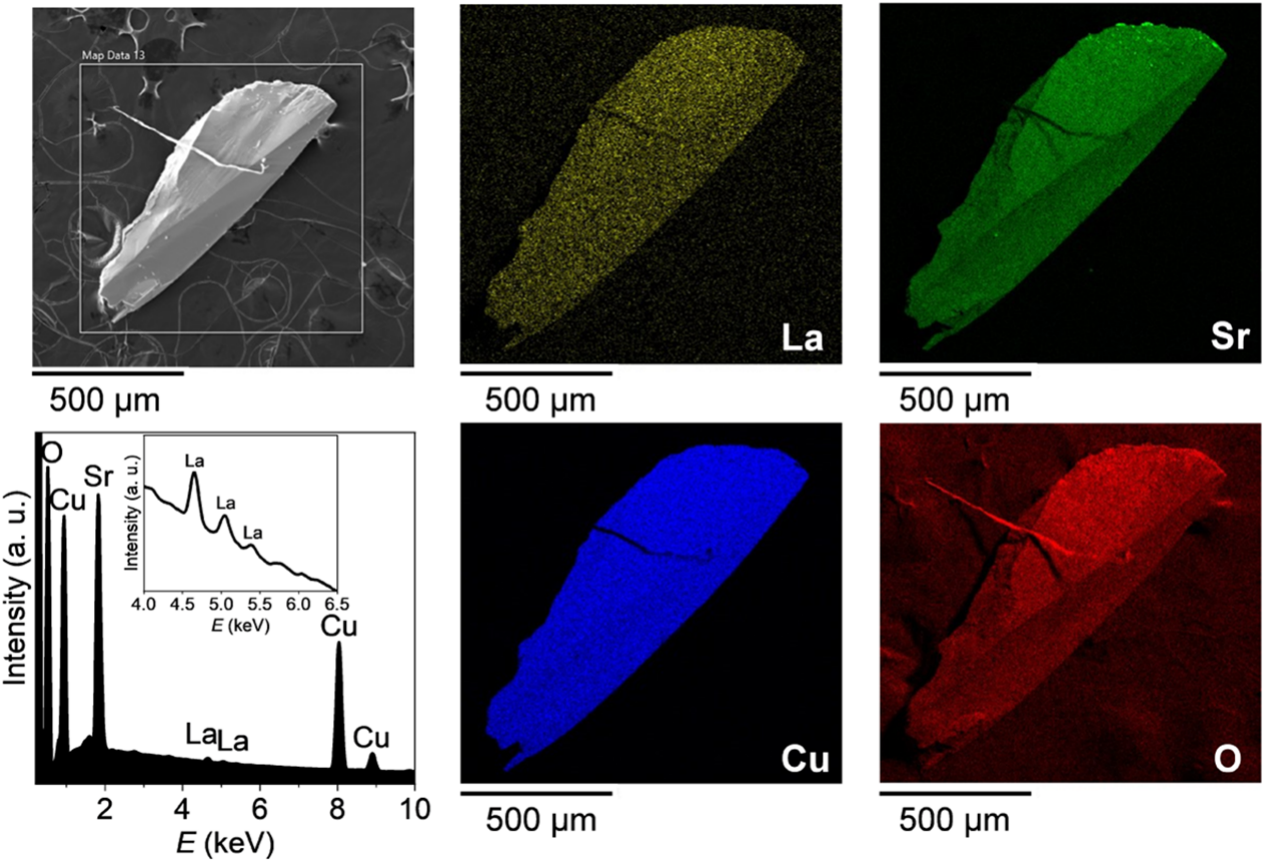
SEM image and EDS elemental mapping analysis of a Sr_1–*x*_La_*x*_Cu_2_(BO_3_)_2_ single crystal with nominal *x* = 0.05 reveal a relatively homogeneous distribution of La within the SrCu_2_(BO_3_)_2_ matrix. The corresponding summed EDS spectrum clearly shows the presence of La emission lines. The inset shows a magnified region, where the La emission lines are clearly seen.

The average semiquantitative atomic percentages of Sr, Cu, O, and La in both undoped and La-doped SrCu_2_(BO_3_)_2_ single crystals, obtained from SEM-EDS point analyses, are summarized in Supporting Information, Table S3. These values are compared with nominal values, i.e., the intended or theoretical amount of dopant defined by the chemical formulas. The table also includes additional ratios such as La/Cu, La/Sr, and Cu/Sr as well as semiquantitative estimates of effective La doping for each nominal concentration. The average La atomic percentage systematically increases with the nominal doping. The effective doping—i.e., the actual amount of dopant incorporated into the crystal structure—reaches approximately 50% of the nominal value for *x* ≤ 0.03 (Supporting Information, Table S3). At higher doping levels (*x* = 0.05, 0.10, and 0.15), the effective doping continues to increase, albeit with smaller increments, reaching ∼20% of the nominal values. Specifically, the effective doping was estimated to be 1.7(3) mol % for *x* = 0.05, 2.0(4) mol % for *x* = 0.10, and 2.7(3) mol % for *x* = 0.15 (Supporting Information, Table S3). While these EDS results confirm La incorporation into the SrCu_2_(BO_3_)_2_ structure, they also indicate that doping is only partial, which emphasizes the challenges of chemical substitutions in this system. This finding aligns well with previous studies.^30,37,38^

### 3.3 Magnetic Susceptibility

Following the successful incorporation of La into the SrCu_2_(BO_3_)_2_ structure, we next measured dc magnetic susceptibility in the 2–300 K temperature region, at a magnetic field of 1 kG. The behavior of the susceptibility curves (Figure 7) is consistent across all the nominal doping concentrations (measured up to *x* = 0.15) and aligns well with previous reports in pristine and doped SrCu_2_(BO_3_)_2_ samples.^35,37,38^ Following the high-temperature Curie–Weiss region, a broad susceptibility maximum at temperature *T*_max_, appears between 15.5 and 18.7 K. The values of *T*_max_ (Table 1) do not show significant changes with *x*, however, they exhibit a small decrease with the increasing nominal doping. At lower temperatures, the susceptibility exhibits a sharp suppression, indicative of the effective spin gap, followed by a low-temperature Curie–like upturn—a hallmark of unpaired Cu^2+^ *S* = 1/2 spins^35,37,38^ —but which also includes a thermally activated contribution and a temperature-independent contribution from the ion cores (described by eq 3). The magnitude of this upturn systematically increases with the nominal La-doping concentration and is most pronounced at *x* = 0.15 (inset, Figure 7). The observed magnetic response is in good agreement with previous studies on La-doped single crystals.^37,38^ The current study also shows that increasing nominal doping leads to a higher concentration of free Cu^2+^ spins in the system, consistent with doping-induced Cu^2+^ spin-dimer breaking. A dimer breaking in Sr-site substituted samples was previously observed and directly probed by μSR in ref^38^.

**Table 1:** Summary of Magnetic Parameters for Sr_1–*x*_La_*x*_Cu_2_(BO_3_)_2_ Single Crystals with Nominal *x* = 0–0.15^a^

SrCu_2_(BO_3_)_2_ nominal doping	*T*_max_ (K)	Effective spin gap, Δ (K)	C’’ (emu K/mol Cu) low-*T* fit 2–3.4 K)	Estimated fraction of free Cu^2+^ spins (%)
*x* = 0	18.4(3)	28.2(3)	1.05(7) · 10^–3^	0.28(2)
*x* = 0.02	17.05(6)	24.7(2)	4.3(2) · 10^–3^	1.15(6)
*x* = 0.03	16.8(2)	23.0(2)	5.4(2) · 10^–3^	1.44(5)
*x* = 0.04	16.68(5)	23.1(3)	5.1(2) · 10^–3^	1.36(5)
*x* = 0.05	16.45(6)	22.1(2)	5.6(2) · 10^–3^	1.49(6)
*x* = 0.10	15.8(2)	20.8(1)	6.9(2) · 10^–3^	1.84(5)
*x* = 0.15	15.8(3)	20.3(4)	9.2(4) ·10^–3^	2.45(11)

a The characteristic temperature *T*_max_ is extracted from Lorentz fits to the magnetic susceptibility in the 15–21 K range. The effective spin gap is obtained from fits to the low temperature susceptibility data (2–6 K) using eq 3. The Curie constant C’ is derived from Curie–Weiss fits to inverse susceptibility data at low temperature (2–3.4 K, Supplementary Information, Figure S5). The estimated fraction of free Cu^2+^ spins is derived from these fits.

**Figure 7:**
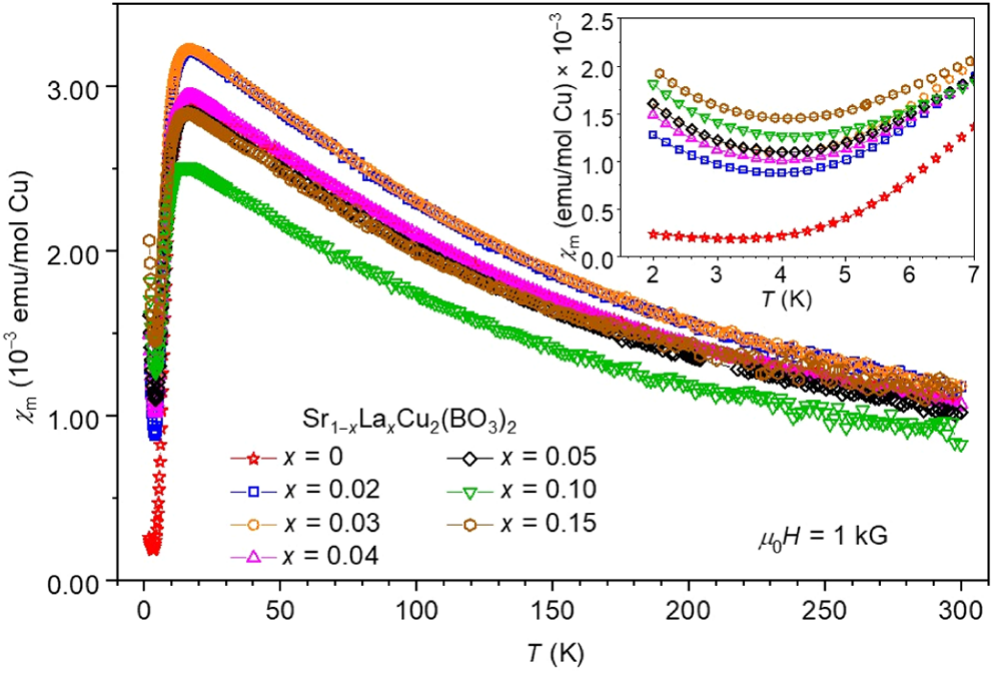
Temperature dependencies of magnetic susceptibility of Sr_1–*x*_La_*x*_Cu_2_(BO_3_)_2_ single crystals with nominal *x* = 0–0.15, measured at μ_0_ *H* = 1 kG. The inset magnifies the low-temperature region, which exhibits the Curie–Weiss upturn, and includes low-temperature fits based on eq 3, performed in the 2–6 K range with θ’ fixed at −0.75 K.

To estimate the evolution of the effective spin gap with La doping, low-temperature magnetic susceptibility data are fitted in the interval 2–6 K according to eq 3:^35^

$$\chi =\frac{C^{\prime }}{T-\theta^{\prime }}+ae^{-\Delta /T}+\chi_{0}$$ 3

The first term represents the spin-1/2 magnetic impurities, characterized by the Curie constant C’ and their weak coupling captured by the Curie–Weiss temperature for the tail, θ’. The second term is the contribution from the antiferromagnetic dimer structure. The third term is a small diamagnetic contribution from the ion cores. The effective spin gap, Δ, is included in the thermally activated contribution. The results of the fits, the C’, θ’, and Δ parameters are presented in Supporting Information, Table S4. Due to a high correlation between the C’ and θ’ parameters, the fits are performed with θ’ fixed at five different values from 0 to −1, with the fits results for θ’ = −0.75 K being chosen as the most representative. The results reveal a gradual closing of the effective spin gap, Δ, from 28.2(3) to 20.3(4) K for nominal doping up to *x* = 0.15 (Table 1) relative to undoped SrCu_2_(BO_3_)_2_. This finding is consistent with the results published in ref^35^, where a similar gap suppression for polycrystalline Sr_0.9_La_0.1_Cu_2_(BO_3_)_2_ from 21.5 to 14.1 K was found. Specifically, the 7.4 K reduction in Δ observed in ref^35^ equals the reduction observed in the current study for the nominal *x* = 0.10 sample.

The concentration of overall dimer-free Cu^2+^ spins in the La-doped samples is determined by fitting the linear 2–3.4 K temperature range of the inverse magnetic susceptibility data to a Curie–Weiss law (Supporting Information, Figure S5) similar to other reports.^41^ The results of the fit (Table 1) show a general increase of the Curie constant C’’ with nominal doping. Specifically, a nominal doping of *x* = 0.10 approximately increases the concentration of dimer-free spins by a factor of ∼7 compared to the undoped crystal, rising from 0.28(2)% for the undoped system to 1.84(5)% for *x* = 0.10, and reaching 2.45(11)% for nominal *x* = 0.15. These values are in good agreement with the effective dopant concentrations obtained from EDS measurements (see Supporting Information, Table S3). Crystals with nominal *x* = 0.04 yield 1.36(5)%, which is about 2.5 times higher than the fraction of impurity spins in optical-floating-zone-grown crystals by Dabkowska et al.^37^ (as reported in ref^38^ ). Note that the concentration of impurity spins observed in this study for La-doped samples is relatively small compared to that reported for Mg doping,^30^ which suggests that La doping is more challenging.

### 3.4 X-Band Electron Spin Resonance Measurements (ESR)

To gain deeper insights into the magnetism of La-doped SrCu_2_(BO_3_)_2_, continuous wave X-band electron spin resonance (ESR) spectroscopy, a local probe technique well suited for detecting paramagnetic states in low-dimensional magnets,^49^ is employed next. This study extends the use of ESR spectroscopy to La-doped SrCu_2_(BO_3_)_2_—a system that has received little attention so far. Previous ESR studies have primarily focused on the undoped system,^48–52^ with only one report on Mg-doped SrCu_2_(BO_3_)_2_.^30^

In this study, temperature-dependent ESR spectra for La-doped single crystals with nominal *x* = 0.02–0.15 were measured down to approximately 4 K, and compared with the ESR data obtained on polycrystalline undoped SrCu_2_(BO_3_)_2_ reported in the literature.^30^ At high temperatures, the ESR spectra across all nominal doping concentrations closely follow the behavior observed in the undoped system (Supporting Information, Figure S6). The main signal, originating from the dimer lattice, broadens with a decrease in temperature, reflecting the development of spin correlations within the Shastry–Sutherland dimer lattice. At low temperatures, an additional component, only weakly observed in the undoped system at *T* ≤ 14 K, emerges in the spectra of doped samples, at *T* ≤ 35 K for nominal *x* = 0.02 and 0.03, and *T* ≤ 50 K for higher nominal concentrations. As the nominal doping increases, this component becomes progressively more pronounced (Supporting Information, Figure S7). Its ESR signal intensity scaling with *x* aligns well with the increase of the Curie–Weiss upturn with nominal doping observed in low-temperature magnetic susceptibility, and further corroborates the presence of intrinsic Cu^2+^ impurities in the doped system as isolated, dimer-free Cu^2+^ spins emerging after the La doping. Furthermore, at the lowest investigated temperatures, *T* ≤ 5.5 K, we observe the emergence of a third, broader component, which can be tentatively associated with the emerging correlations between dimer-free Cu^2+^ spins when their concentration increases.

For a quantitative analysis, the ESR spectra are fitted using up to three Lorentzian components depending on the temperature range (Figure 8a).

**Figure 8:**
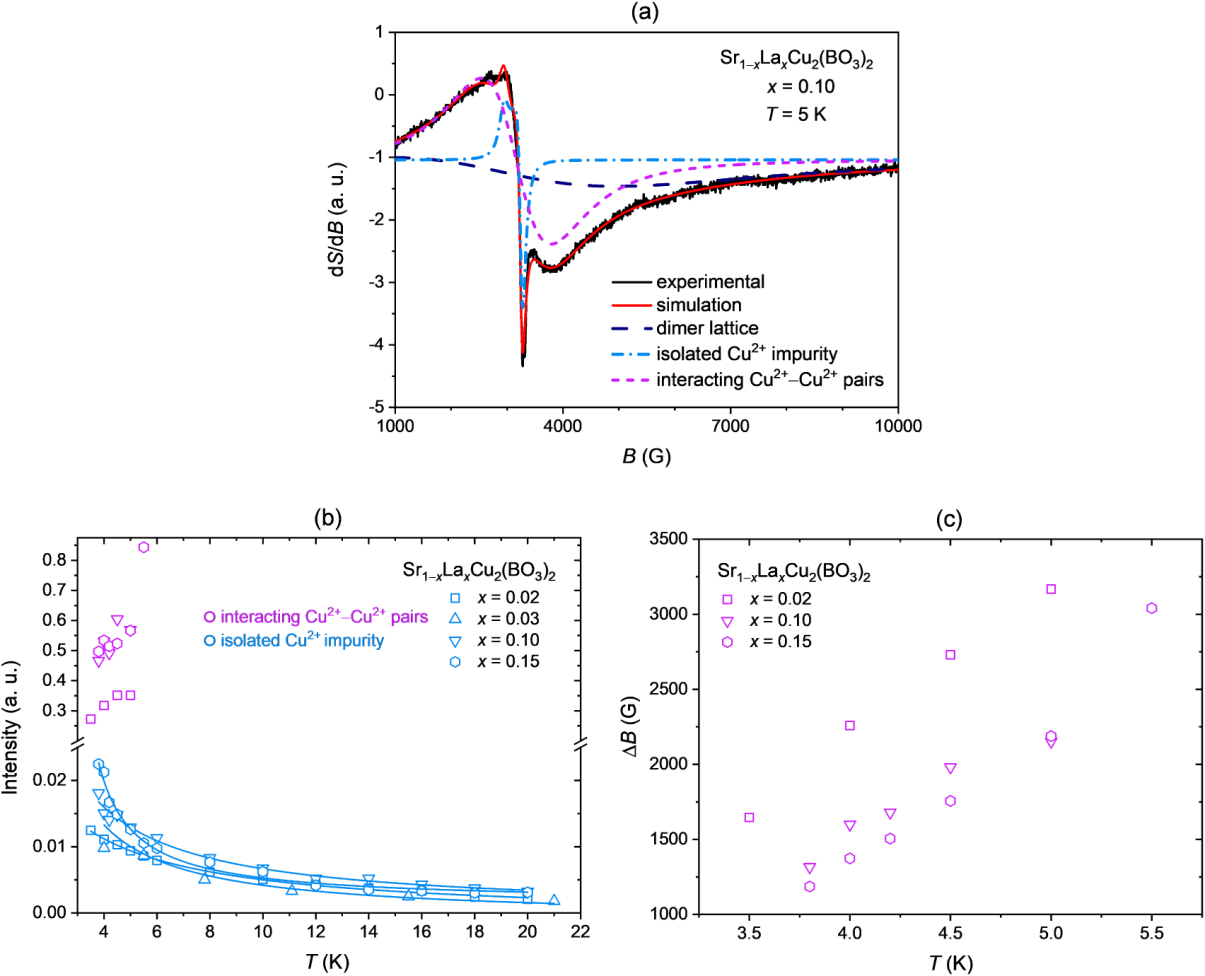
(**a**) X-band ESR spectrum of several Sr_1−*x*_La_*x*_Cu_2_(BO_3_)_2_ single crystals with *x* = 0.10 measured at 5 K. The corresponding simulation (red line) is composed of three Lorentzian components: (i) the broad dimer lattice signal (dashed dark blue lines), (ii) an anisotropic component attributed to the isolated Cu^2+^ impurity component (short dash-dot light-blue lines), and (iii) interacting Cu^2+^–Cu^2+^ pairs that emerge between dimer-free Cu^2+^ spins (short dashed violet lines). (**b**) Temperature dependence of the signal intensities of the isolated Cu^2+^ impurity component and the interacting Cu^2+^–Cu^2+^ pair component for selected nominal doping levels. (**c**) Temperature dependence of the line width Δ*B* of the interacting Cu^2+^–Cu^2+^ pair component (iii) for *x* = 0.02, 0.10, and 0.15, showing a monotonic decrease with the decreasing temperature for all doping levels, consistent with the presence of antiferromagnetic exchange interactions between dimer-free Cu^2+^ spins.

At high temperatures, only a broad line attributed to the Cu^2+^ dimer lattice is required. Below 50 K, an additional anisotropic component with axial *g*-factor anisotropy is included to account for the isolated Cu^2+^ intrinsic impurities. Here, *g*_∥_ and *g*_⊥_ represent the two *g* factor eigenvalues parallel and perpendicular to the crystallographic *c* axis, respectively. A corresponding uniaxial anisotropy is also applied to the line width, defined by Δ*B*_∥_ and Δ*B*_⊥_ for the field orientations along and perpendicular to the *c* axis, respectively. Below 5.5 K, a third broad isotropic Lorentzian line has to be introduced, which is tentatively assigned to the onset of emerging correlations between unpaired Cu^2+^ spins. The intensities of all three components, resulting from the fits, are normalized to the room temperature value for all samples.

At room temperature, the main dimer-lattice component is centered around *g* ≈ 2.1. The line width, Δ*B*, remains similar across all nominal doping concentrations, ranging from 1350 to 1550 G (Supporting Information, Figure S8a). This matches the corresponding line width and *g*-factor values reported in the literature for undoped SrCu_2_(BO_3_)_2_ powder.^49^ Upon cooling, the line width of this component increases monotonically, saturating near 10 K at values between 4400 and 5100 G. This broadening reflects the gradual development of short-range antiferromagnetic correlations in the dimer lattice and becomes less dominant at low temperatures as the isolated Cu^2+^ impurity component begins to dominate the ESR response. The intensity of this component increases with decreasing temperature (Figure 8b), following a 1/*T* dependence across all nominal doping concentrations. The data are fitted using the Curie–Weiss law, yielding a Curie–Weiss temperature θ’ between −2 and +2.5 K, effectively close to zero, confirming that the corresponding Cu^2+^ moments are only weakly interacting and supports their identification as isolated, dimer-free spins. Compared to the isolated Cu^2+^ impurity component, the intensities of the broader component assigned to liberated but interacting Cu^2+^–Cu^2+^ pairs, introduced in the low-temperature regime (3.5–5.5 K), are significantly higher across all doping concentrations (Figure 8b). Specifically, the interacting Cu^2+^–Cu^2+^ pair component intensity exceeds the isolated Cu^2+^ impurity component intensity by factors ranging from approximately 10 to 40, highlighting the emergence of magnetic correlations between dimer-free Cu^2+^ spins. These correlations become increasingly more prominent with a higher nominal doping. For instance, samples with *x* = 0.10 and 0.15 exhibit stronger intensities than *x* = 0.02, suggesting that higher nominal doping results in more pronounced Cu^2+^–Cu^2+^ interactions. Moreover, at higher nominal doping, these correlated states persist to higher temperatures, as evidenced by the interacting Cu^2+^–Cu^2+^ pair component up to 5.5 K for the *x* = 0.15 sample, compared to only up to 5 K for *x* = 0.02 and 0.10. This trend is further corroborated by the temperature dependence of the line width, Δ*B*, of the interacting Cu^2+^–Cu^2+^ pair component.

Overall, Δ*B* shows a systematic decrease with increasing doping concentration, as illustrated at 4 K (Figure 8c), where the line width reaches its highest value of 2260 G for *x* = 0.02, decreases to 1600 G for *x* = 0.10, and attains its lowest value of 1370 G for *x* = 0.15. Additionally, for each doping concentration individually, the line width exhibits a consistent monotonic decrease with decreasing temperature, indicative of the antiferromagnetic nature of the exchange interactions between dimer-free Cu^2+^ spins.^53,54^ The *g*-factor of the interacting Cu^2+^–Cu^2+^ pair component remains constant at approximately 2.12 for all doping levels across the whole temperature range (Supporting Information, Figure S8b).

Due to the large number of fitting parameters and their strong correlations, the line width and *g*-factor components of the isolated Cu^2+^ impurity component were partially constrained during analysis. Specifically, Δ*B*_∥_ was fixed across all data sets to ensure stable fitting. Throughout most of the temperature range, Δ*B*_⊥_ remained relatively constant, falling within the interval 150–200 G (Supporting Information, Figure S8c). However, below 5.5 K—coinciding with the emergence of component (iii)—a monotonic increase in Δ*B*_⊥_ was observed, providing an additional indication of developing correlations between dimer-free Cu^2+^ spin pairs. Similarly, *g*_∥_ was fixed for all doping concentrations except for *x* = 0.02, where a gradual decrease was detected below 6 K. The perpendicular component, *g*_⊥_, remained effectively constant around 2.065 across the full temperature range for all samples (Supporting Information, Figure S8d).

In contrast to the general trends observed across nominal *x* = 0.02–0.15, the spectra for nominal *x* = 0.05 deviate substantially for both the isolated Cu^2+^ impurity component and the interacting Cu^2+^–Cu^2+^ pair component (Supporting Information, Figure S9a). Contrary to the rest of the samples, this sample exhibits a clear and distinct *g* factor anisotropy of the isolated Cu^2+^ impurity component, which allows a clear and unambiguous determination of both Δ*B*_∥_ and Δ*B*_⊥_. These line width components exhibit a substantial increase with decreasing temperature (Supporting Information, Figure S9b), exceeding the modest changes in the line width observed for the rest of the samples, which occur only below 5.5 K. The intensity of the isolated dimer-free Cu^2+^ component behaves similarly to the other samples (Supporting Information, Figure S9c), following a 1/*T* dependence and yielding a Curie–Weiss temperature, θ′, close to 0. In contrast, the interacting Cu^2+^–Cu^2+^ pair component for *x* = 0.05 shows further anomalies as its Δ*B* remains constant at a significantly lower value of approximately 750 G (Supporting Information, Figure S9d), deviating from the decreasing trend observed in the other samples.

In summary, X-band ESR measurements across all nominal La-doping concentrations reveal multiple components in the spectra: a broad signal from the spin dimer lattice, an anisotropic contribution, which denotes the presence of isolated Cu^2+^ impurities, and an additional low-temperature component arising from dimer-free Cu^2+^–Cu^2+^ interactions. The evolution of the latter component below 5.5 K suggests antiferromagnetic correlations between interacting dimer-free Cu^2+^ spin pairs, which are enhanced with increasing doping.

### 3.5 High Magnetic Field Electron Spin Resonance Measurements

Magnetic susceptibility provides an indirect estimate of the spin gap or so-called “effective” spin gap through fitting low-temperature data. This effective gap represents an approximate or averaged measure of the gapped behavior of magnetic susceptibility and can be affected by the presence of in-gap states. As a result, even a modest density of localized spins or in-gap states can lower the effective gap as deduced from susceptibility fits while leaving the intrinsic singlet–triplet gap probed by the high-field ESR unchanged. The intrinsic or actual spin gap, defined as the excitation energy required for singlet–triplet excitation, can be directly determined using microscopic probes such as high-field ESR or inelastic neutron scattering. The first reported spin gap for SrCu_2_(BO_3_)_2_ was Δ = 34 K in 1999.^20^ In the present study, to obtain a more precise measurement of the spin gap for La-doped SrCu_2_(BO_3_)_2_, a group of stacked single crystals with nominal *x* = 0.03 was analyzed using high-field ESR at multiple frequencies up to 500 GHz and magnetic fields up to 160 kG (Figure 9a).

**Figure 9:**
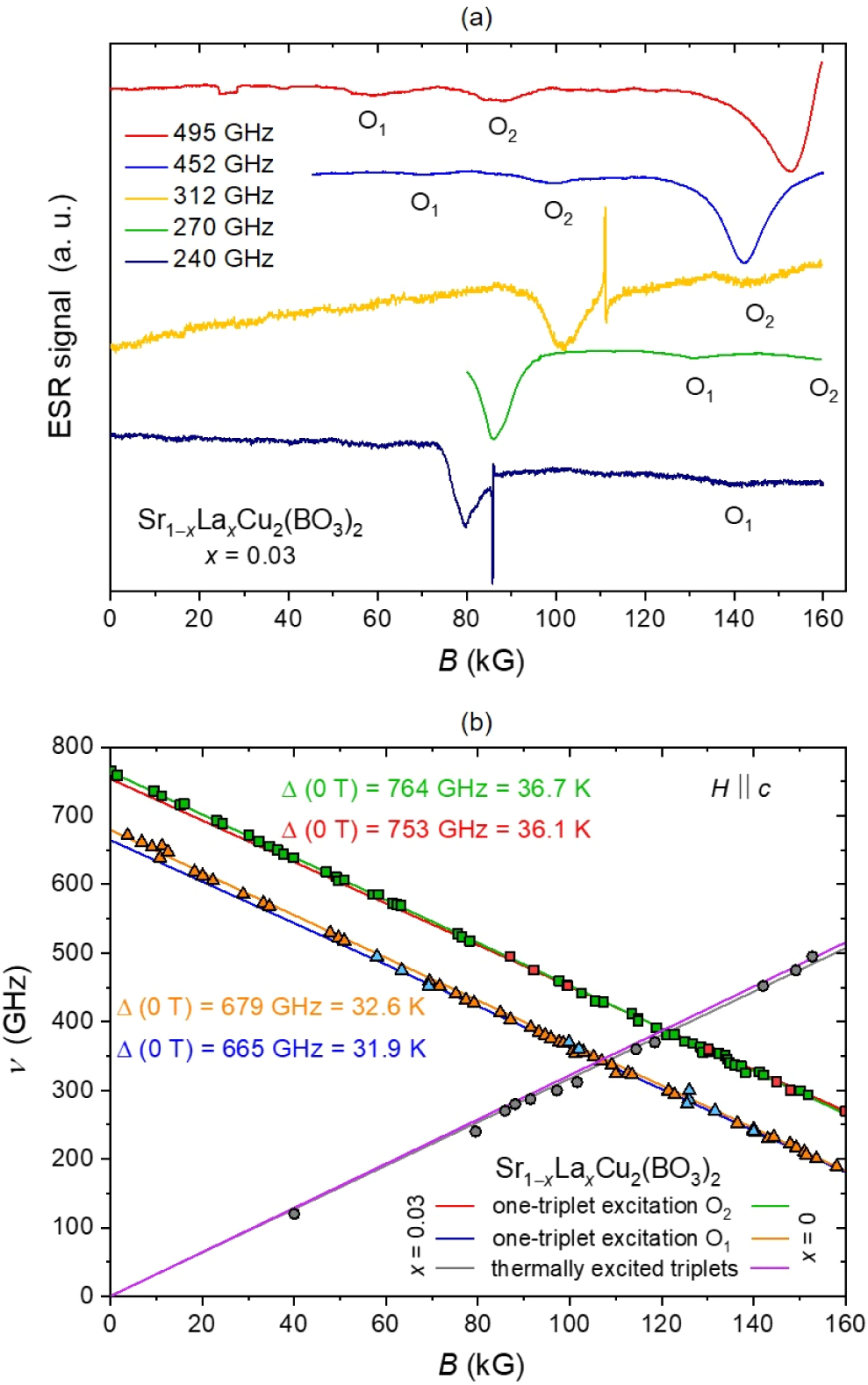
(**a**) Low-temperature high-field ESR spectra of stacked Sr_1−*x*_La_*x*_Cu_2_(BO_3_)_2_ single crystals with *x* = 0.03 at selected frequencies. (**b**) The corresponding frequency-field diagram (*x* = 0.03) at *T* = 1.7–2.9 K for H ∥ c, comparing thermally excited triplets and two sets of one-triplet excitations, O_1_ and O_2_, with literature data for the undoped system (*x* = 0), showing a respective 0.6 and 0.7 K reduction of the spin gap induced by doping.

The main signals previously observed within this frequency-field range for the undoped system are two low-lying singlet–triplet excitations, O_1_ and O_2_, as well as paramagnetic signals attributed to thermally excited triplets.^55^

The frequency–field diagram of the undoped system in the orientation *H* ∥ *c*, reveals zero-field energy gaps of 764(2) GHz and 679(2) GHz for O_1_ and O_2_, respectively, corresponding to 36.7 and 32.6 K.^55^ Comparing these values to those obtained experimentally from the frequency-field diagram of the *x* = 0.03 doped crystals (Figure 9b), the spin gaps are only found to be slightly reduced to 753 GHz (36.1 K) for O_1_ and 665 GHz (31.9 K) for O_2_. These represent decreases of only 0.6 and 0.7 K compared to the undoped system, indicating that the effect of nominal *x* = 0.03 La doping has a minimal effect on the actual spin gap of SrCu_2_(BO_3_)_2_. Since the O_1_ and O_2_ excitations reflect the intrinsic spin dynamics of the dimerized Cu^2+^ lattice and do not account for the presence of intrinsic dimer-free Cu^2+^ impurities or their local magnetic interactions, the observed minimal reduction in the spin gap indicates that the collective spin excitations within the main dimer lattice remain largely unaffected by nominal *x* = 0.03 doping. Consequently, the notable decrease in the effective spin gap of 4.7 K, determined from the magnetic susceptibility, is primarily influenced by localized magnetic moments associated with intrinsic impurity sites. Capturing additional excitations caused by intrinsic impurities and their interactions through high-field ESR will require measurements at higher frequencies and/or stronger magnetic fields as well as examination of the remaining samples covering the entire nominal doping range.

## 4 Conclusions

This study introduces a facile flux method to grow La-doped SrCu_2_(BO_3_)_2_ single crystals with lateral sizes of up to 3 mm. A large range of La nominal doping concentrations, *x* = 0.02, 0.03, 0.04, 0.05, 0.10, and 0.15 in Sr_1–*x*_La_*x*_Cu_2_(BO_3_)_2_, is systematically explored. Doping-induced structural changes are subtle but systematic, causing a decrease in unit cell parameters with increasing La concentration. The successful incorporation of La into the parent SrCu_2_(BO_3_)_2_ structure is corroborated by EDS-SEM analysis. The semiquantitative evaluation of effective doping concentrations reveals that La incorporation is <50% of the nominal values, reaching up to 2.7(3) mol % for nominal *x* = 0.15, highlighting the inherent difficulty of doping SrCu_2_(BO_3_)_2_.

In addition to the structural modifications, La doping also induces systematic effects on the magnetism of SrCu_2_(BO_3_)_2_. Compared to the undoped system, impurity-induced in-gap states introduced by La doping lead to a progressive reduction of the effective spin gap extracted from magnetic susceptibility measurements from 28.2 K for *x* = 0 to 20.3 K for nominal La doping of *x* = 0.15. Additionally, X-band ESR measurements reveal a systematic increase in the fraction of the dimer-free Cu^2+^ (*S* = 1/2) spins with nominal doping, along with the emergence of antiferromagnetic correlations between dimer-free Cu^2+^ spin pairs below 5.5 K. High-field ESR measurements further indicate that at low doping levels, the effective spin gap reduction observed in susceptibility primarily arises from these intrinsic impurity spins, while the collective spin dynamics of the Cu^2+^ dimer lattice remain largely intact. Despite these systematic changes in structure and magnetism, no superconductivity is observed across the entire range of La doping studied.

The present findings underscore the potential of flux growth for La doping in SrCu_2_(BO_3_)_2_, providing a more efficient and accessible alternative to optical floating zone growth with the possibility of extending this method to other dopants.

## Supplementary Material



## Data Availability

The data that support the findings of this study are openly available on Zenodo at: https://pubs.acs.org/doi.org/10.5281/zenodo.17952985.

## Notes

Rietveld refinement results, average semiquantitative atomic % values of constituent elements in Sr_1–*x*_La_*x*_Cu_2_(BO_3_)_2_ single crystals, determined by EDS-SEM, compared to nominal values, low-temperature fitting results of magnetic susceptibility, and low-temperature inverse susceptibility fits in the interval 2–3.4 K, high- and low-temperature X-band ESR spectra, and the development of multiple parameters for three components, derived from the simulations, for selected nominal doping concentrations (PDF)

## References

[1] Bednorz J. G., Müller K. A. (1986). Possible high *T*
_c_ superconductivity
in the Ba-La-Cu-O system. Z. Phys. B.

[2] Introduction to Frustrated Magnetism, Lacroix, C. ; Mendels, P. ; Mila, F. , Eds.; Springer-Verlag: Berlin, Heidelberg, 2011.

[3] Anderson P. W. (1973). Resonating
valence bonds: A new kind of insulator?. Mater.
Res. Bull..

[4] Anderson P. W. (1987). The resonating
valence bond state in La_2_CuO_4_ and superconductivity. Science.

[5] Hase M., Terasaki I., Uchinokura K. (1993). Observation
of the spin-Peierls transition
in linear Cu^2+^ (spin1/2) chains in an inorganic compound
CuGeO_3_. Phys. Rev. Lett..

[6] Buyers W. J. L., Morra R. M., Armstrong R. L., Hogan M. J., Gerlach P., Hirakawa K. (1986). Experimental evidence
for the Haldane gap in a spin-1
nearly isotropic, antiferromagnetic chain. Phys.
Rev. Lett..

[7] Azuma M., Hiroi Z., Takano M., Ishida K., Kitaoka Y. (1994). Observation
of a spin gap in SrCu_2_O_3_ comprising spin-1/2
quasi-one-dimensional two-leg ladders. Phys.
Rev. Lett..

[8] Taniguchi S., Nishikawa T., Yasui Y., Kobayashi Y., Sato M., Nishioka T., Kontani M., Sano K. (1995). Spin Gap Behavior
of *S* = 1/2 Quasi-Two-Dimensional System CaV_4_O_9_. J. Phys. Soc. Jpn..

[9] Kageyama H., Kitano T., Oba N., Nishi M., Nagai S., Hirota K., Viciu L., Wiley J. B., Yasuda J., Baba Y. (2005). Spin-Singlet
Ground State in Two-Dimensional *S* = 1/2 Frustrated
Square Lattice: (CuCl)­LaNb_2_O_7_. J. Phys. Soc. Jpn..

[10] Calder S., Pajerowski D. M., Stone M. B., May A. F. (2018). Spin-Gap and Two-Dimensional
Magnetic Excitations in Sr_2_IrO_4_. Phys. Rev. B.

[11] Smith R. W., Keszler D. A. (1991). Synthesis, structure, and properties
of the orthoborate
SrCu_2_(BO_3_)_2_. J. Solid State Chem..

[12] Kageyama H., Yoshimura K., Stern R., Mushnikov N. V., Onizuka K., Kato M., Kosuge K., Slichter C. P., Goto T., Ueda Y. (1999). Exact Dimer Ground State and Quantized
Magnetization Plateaus in the Two-Dimensional Spin System SrCu_2_(BO_3_)_2_. Phys.
Rev. Lett..

[13] Miyahara S., Ueda K. (1999). Exact Dimer Ground State of the Two Dimensional Heisenberg Spin System
SrCu_2_(BO_3_)_2_. Phys. Rev. Lett..

[14] Sriram S. B., Sutherland B. (1981). Exact ground state of a quantum mechanical
antiferromagnet. Phys. B+C.

[15] Knetter C., Bühler A., Müller-Hartmann E., Uhrig G. S. (2000). Dispersion
and Symmetry of Bound States in the Shastry–Sutherland Model. Phys. Rev. Lett..

[16] Dorier J., Schmidt K. P., Mila F. (2008). Theory of Magnetization
Plateaux
in the Shastry–Sutherland Model. Phys.
Rev. Lett..

[17] Koga A., Kawakami N. (2000). Quantum Phase Transitions
in the Shastry-Sutherland
Model for SrCu_2_(BO_3_)_2_. Phys. Rev. Lett..

[18] Yang J., Sandvik A. W., Wang L. (2022). Quantum criticality
and spin liquid
phase in the Shastry Sutherland model. Phys.
Rev. B.

[19] Viteritti L. L., Rende R., Parola A., Goldt S., Becca F. (2025). Transformer
Wave Function for Two-Dimensional Frustrated Magnets: Emergence of
a Spin-Liquid Phase in the Shastry–Sutherland Model. Phys. Rev. B.

[20] Nojiri H., Kageyama H., Onizuka K., Ueda Y., Motokawa M. (1999). Direct Observation
of the Multiple Spin Gap Excitations in Two-Dimensional Dimer System
SrCu_2_(BO_3_)_2_. J. Phys. Soc. Jpn..

[21] Kageyama H., Onizuka K., Yamauchi T., Ueda Y., Hane S., Mitamura H., Goto T., Yoshimura K., Kosuge K. (1999). Anomalous Magnetizations in Single Crystalline SrCu_2_(BO_3_)_2_. J. Phys.
Soc. Jpn..

[22] Kageyama H., Nishi M., Aso N., Onizuka K., Yosihama T., Nukui K., Kodama K., Kakurai K., Ueda Y. (2000). Direct evidence
for the localized single-triplet excitations and the dispersive multitriplet
excitations in SrCu_2_(BO_3_)_2_. Phys. Rev. Lett..

[23] Onizuka K., Kageyama H., Narumi Y., Kindo K., Ueda Y., Goto T. (2000). 1/3 Magnetization Plateau in SrCu_2_(BO_3_)_2_ Stripe Order of Excited Triplets-. J. Phys. Soc. Jpn..

[24] Haravifard S., Graf D., Feiguin A. E., Batista C. D., Lang J. C., Silevitch D. M., Srajer G., Gaulin B. D., Dabkowska H. A., Rosenbaum T. F. (2016). Crystallization of spin superlattices with pressure
and field in the layered magnet SrCu_2_(BO_3_)_2_. Nat. Commun..

[25] Boos C., Crone S. P. G., Niesen I. A., Corboz P., Schmidt K. P., Mila F. (2019). Competition Between Intermediate Plaquette Phases in SrCu_2_(BO_3_)_2_ Under Pressure. Phys. Rev. B.

[26] Zayed M. E., Rüegg C., Larrea J., Läuchli A. M., Panagopoulos C., Saxena S. S., Ellerby M., McMorrow D. F., Klotz T., Klotz S. (2017). 4-Spin Plaquette Singlet
State in the Shastry–Sutherland Compound SrCu_2_(BO_3_)_2_. Nat. Phys..

[27] Guo J., Sun G., Zhao B., Wang L., Hong W., Sidorov V. A., Ma N., Wu Q., Li S. (2020). Quantum Phases of SrCu_2_(BO_3_)_2_ from High-Pressure Thermodynamics. Phys. Rev. Lett..

[28] Jiménez J. L., Crone S. P. G., Fogh E., Zayed M. E., Lortz R., Pomjakushina E., Conder K., Läuchli A. M., Weber L., Wessel S. (2021). A Quantum Magnetic Analogue
to the Critical Point of Water. Nature.

[29] Cui Y., Liu L., Lin H., Wu K.-H., Hong W., Liu X., Li C., Hu Z., Xi N., Li S. (2023). Proximate
deconfined quantum critical point in SrCu_2_(BO_3_)_2_. Science.

[30] Šibav L., Gosar Ž., Knaflič T., Jagličić Z., King G., Nojiri H., Arčon D., Dragomir M. (2024). HigherMagnesiumDoping Effects on
the Singlet Ground
State of the Shastry-Sutherland SrCu_2_(BO_3_)_2_. Inorg. Chem..

[31] Shastry B. S., Kumar B. (2002). SrCu_2_(BO_3_)_2_: A unique Mott Hubbard
insulator. Prog. Theor. Phys. Suppl..

[32] Liu J., Trivedi N., Lee Y., Harmon B. N., Schmalian J. (2007). Quantum Phases
in a Doped Mott Insulator on the Shastry-Sutherland Lattice. Phys. Rev. Lett..

[33] Yang B., Kim Y. B., Yu J., Park K. (2008). Doped valence-bond
solid and superconductivity on the Shastry-Sutherland lattice. Phys. Rev. B.

[34] Norrestam R., Carlson S., Kritikos M., Sjödin A. (1994). Synthetic
Structural, and Magnetic Studies of Strontium Copper­(II) Borates with
the Composition Sr_1‑*x*
_
*M_x_
*CU_2_(BO_3_)_2_, *M* = Ba or Ca. J. Solid State Chem..

[35] Liu G. T., Luo J. L., Wang N. L., Jing X. N., Jin D., Xiang T., Wu Z. H. (2005). Doping
effects on the two-dimensional
spin dimer compound SrCu_2_(BO_3_)_2_. Phys. Rev. B.

[36] Choi K.-Y., Pashkevich Y., Lamonova K., Kageyama H., Ueda Y., Lemmens P. (2003). Strong anharmonicity and spin-phonon coupling in the
quasi-two-dimensional quantum spin system Sr_1—*x*
_Ba*
_x_
*Cu_2_(BO_3_)_2_. Phys. Rev. B.

[37] Dabkowska H. A., Dabkowski A. B., Luke G. M., Dunsiger S. R., Haravifard S., Cecchinel M., Gaulin B. D. (2007). Crystal growth and
magnetic behaviour
of pure and doped SrCu_2_(^11^BO_3_)_2_. J. Cryst. Growth.

[38] Aczel A. A., MacDougall G. J., Rodriguez J. A., Luke G. M., Russo P. L., Savici A. T., Uemura Y. J., Dabkowska H. A., Wiebe C. R., Janik J. A., Kageyama H. (2007). Impurity-induced singlet
breaking in SrCu_2_(BO_3_)_2_. Phys. Rev. B.

[39] Liu G. T., Luo J. L., Guo Y. Q., Su S. K., Zheng P., Wang N. L., Jin D., Xiang T. (2006). In-plane substitution
effect on the magnetic properties of the two-dimensional spin-gap
system SrCu_2_(BO_3_)_2_. Phys. Rev. B.

[40] Haravifard S., Dunsinger S. R., El Shawish S., Gaulin B. D., Dabkowska H. A., Telling M. T. F., Perring T. G., Bonča J. (2006). In-gap Spin
Excitations and Finite Triplet Lifetimes in the Dilute Singlet Ground
State System SrCu_2—x_Mg*
_x_
*(BO_3_)_2_. Phys. Rev. Lett..

[41] Shi Z., Steinhardt W., Graf D., Corboz P., Weickert F., Harrison N., Jaime M., Marjerrison C., Dabkowska H. A., Mila F., Haravifard S. (2019). Emergent bound
states and impurity pairs in chemically doped Shastry-Sutherland system. Nat. Commun..

[42] Rahemtulla A., King G., Gomez A., Appathurai N., Leontowich A. F. G., Castle R., Burns N., Kim C.-Y., Moreno B., Kycia S. (2025). The High Energy Diffraction Beamline
at the Canadian Light Source. J. Synchrotron
Radiat.

[43] Toby B. H., Von Dreele R. B. (2013). GSAS-II: the genesis of a modern open-source all purpose
crystallography software package. J. Appl. Crystallogr..

[44] Zvyagin S. A., Krzystek J., van Loosdrecht P. H.
M., Dhalenne G., Revcolevschi A. (2004). Highfield ESR study of the dimerized-incommensurate
phase transition in the spin-Peierls compound CuGeO_3_. Phys. B.

[45] Hill, J. M. Doping experiments on low-dimensional oxides and a search for unusual magnetic properties of MgAlB_14_ . Ph.D. dissertation; Iowa State University: Ames, Iowa, 2002.

[46] Maltsev V., Leonyuk N., Koporulina E., Dorokhova G. (2004). Flux growth
and morphology of SrCu_2_(BO_3_)_2_ crystals. J. Cryst. Growth.

[47] Maltsev V., Leonyuk N., Szymczak R. (2005). A new advance
in crystal growth of
two-dimensional strontium cuprate–borate. J. Cryst. Growth.

[48] Zorko, A. Study of one- and two-dimensional magnetic systems with spin-singlet ground state. Ph.D. dissertation; University of Ljubljana: Slovenia, 2004.

[49] Zorko A., Arčon D., Lappas A., Giapintzakis J. (2001). Near critical
behavior in the two-dimensional spin-gap system SrCu_2_(BO_3_)_2_. Phys. Rev. B.

[50] Zorko A., Arčon D., van Tol H., Brunel L. C., Kageyama H. (2004). X-band ESR
determination of Dzyaloshinsky-Moriya interaction in the two-dimensional
SrCu_2_(BO_3_)_2_ system. J. Magn. Magn. Mater..

[51] Zorko A., Arčon D., Nuttall C. J., Lappas A. (2004). X-band ESR study of
the 2D spin-gap system SrCu_2_(BO_3_)_2_. J. Magn. Magn. Mater..

[52] Zorko A., Arčon D., Kageyama H., Lappas A. (2004). Magnetic anisotropy
of the SrCu_2_(BO_3_)_2_ system as revealed
by X-band ESR. Appl. Magn. Reson..

[53] Kubo R., Tomita K. (1954). A general theory of
magnetic resonance absorption. J. Phys. Soc.
Jpn..

[54] Bencini, A. ; Gatteschi, D. Electron Paramagnetic Resonance of Exchange Coupled Systems; Springer-Verlag: Berlin, Heidelberg, NY, 1990.

[55] Nojiri H., Kageyama H., Ueda Y., Motokawa M. (2003). ESR Study on the Excited
State Energy Spectrum of SrCu_2_(BO_3_)_2_—A Central Role of Multiple-Triplet Bound States. J. Phys. Soc. Jpn..

