# Supplementary material for: Influence of La Doping on the Magnetic Properties of the Two-Dimensional Spin-Gapped System SrCu2(BO3)2
Source: Inorg Chem. 2025 Dec 29;65(1):291–302. doi: 10.1021/acs.inorgchem.5c04249 (PMC12801323; doi:10.1021/acs.inorgchem.5c04249)
Supplement: Supplementary file 1 [file ic5c04249_si_001.pdf]

# SUPPLEMENTARY INFORMATION FILE

## Influence of La-doping on the magnetic properties of the two-dimensional spin-gapped system $SrCu_2(BO_3)_2$


Lia Šibav[1,2], Tilen Knaflič[1], Graham King[3], Zvonko Jagličić[4,5], Maja Koblar[1], Kirill Povarov[6], Sergei Zvyagin[6], Denis Arčon[1,7], and Mirela Dragomir[1,2*]

[1] Jožef Stefan Institute, Jamova cesta 39, 1000 Ljubljana, Slovenia
[2] Jožef Stefan International Postgraduate School, Jamova cesta 39, 1000 Ljubljana, Slovenia
[3] Canadian Light Source, 44 Innovation Blvd, Saskatoon, SK S7N 2V3, Canada
[4] Institute of Mathematics, Physics and Mechanics, Jadranska ulica 19, 1000 Ljubljana, Slovenia
[5] Faculty of Civil and Geodetic Engineering, University of Ljubljana, Jamova cesta 2, 1000 Ljubljana, Slovenia
[6] Dresden High Magnetic Field Laboratory (HLD-EMFL) and Würzburg-Dresden Cluster of Excellence ct.qmat, Helmholtz-Zentrum Dresden-Rossendorf, 01328 Dresden, Germany
[7] Faculty of Mathematics and Physics, University of Ljubljana, Jadranska ulica 19, 1000 Ljubljana, Slovenia

*Corresponding author: mirela.dragomir@ijs.si

## Table of Contents

**Table S1** Room-temperature structural parameters extracted from Rietveld refinement analyses of synchrotron PXRD data collected on crushed $Sr_{1-x}La_xCu_2(BO_3)_2$ single crystals of all studied nominal doping concentrations, $x$ = 0.02−0.15, compared with polycrystalline undoped $SrCu_2(BO_3)_2$. The refinements were performed in the $I\bar{4}2m$ space group. ........ S3

**Figure S1** Photographs of the blue La-doped $SrCu_2(BO_3)_2$ (in mol%) single crystals grown in this study........ S3

**Figure S2** Rietveld refinement profiles of synchrotron XRD data (λ = 0.3502 Å) collected on crushed $Sr_{1-x}La_xCu_2(BO_3)_2$ single crystals with selected nominal doping concentrations (**a**) $x$ = 0.02, (**b**) 0.05, (**c**) 0.10, and (**d**) 0.15, collected at room temperature. The refinements were performed using the $I\bar{4}2m$ space group. ........ S4

**Table S2** Detailed results of Rietveld refinement analyses of synchrotron PXRD data collected on crushed $Sr_{1-x}La_xCu_2(BO_3)_2$ single crystals of selected nominal doping concentrations $x$ = 0.02, 0.05, 0.10 and 0.15. The data were refined using the $I\bar{4}2$m space group. ........ S5

**Table S3** The average semi-quantitative atomic % values of Sr, Cu, O and La in $Sr_{1-x}La_xCu_2(BO_3)_2$ single crystals with nominal $x$ = 0−0.15, followed by La/Cu, La/Sr and Cu/Sr ratios, compared to the nominal values. The results were determined from SEM-EDS point analyses (20 points for each doping concentration) performed on the crystals. Due to the low relative atomic mass of boron, it could not be detected by EDS and was therefore omitted from the semi-quantitative analysis. ........ S6

**Figure S3** An SEM image and EDS elemental mapping analysis of a $Sr_{1-x}La_xCu_2(BO_3)_2$ single crystal with nominal $x$ = 0.04 (**a**) and 0.10 (**b**) reveal a relatively homogeneous distribution of La within the $SrCu_2(BO_3)_2$ matrix. Few $LaBO_3$ impurity particles are observed on the surface. The corresponding EDS sum spectra clearly show the presence of La emission lines for both a) and b) samples........ S7

**Figure S4** An SEM image (left) and EDS elemental mapping analysis (right) of a $Sr_{1-x}La_xCu_2(BO_3)_2$ single crystal with nominal $x$ = 0.15, showing: (**a**) the presence of $LaBO_3$ impurity particles on the crystal surface and (**b**) the presence of both CuO and $LaBO_3$ impurity particles on the crystal surface. ........ S8

**Table S4** Results of the low-temperature magnetic susceptibility data fits (2−6 K range) for $Sr_{1-x}La_xCu_2(BO_3)_2$ single crystals with nominal $x$ = 0−0.15 (Equation 3 in the main text). Five fixed θ' values were used due to its high correlation with C'. ........ S9

**Figure S5** Inverse magnetic susceptibility data for $Sr_{1-x}La_xCu_2(BO_3)_2$ single crystals with nominal $x$ = 0–0.15, displaying low-temperature Curie–Weiss fits performed in the 2–3.4 K interval to estimate intrinsic $Cu^{2+}$ ($S$ = ½) impurities. ........ S10

**Figure S6** X-band ESR spectra of $Sr_{1-x}La_xCu_2(BO_3)_2$ single crystals with nominal (**a**) $x$ = 0.02, (**b**) 0.03, (**c**) 0.04, (**d**) 0.05, (**e**) 0.10 and (**f**) 0.15, measured as quasi-polycrystalline samples at temperatures 40, 70, 110, 170 and 250 K, which show the dominant signal of the dimer lattice at high temperatures.. ........ S11

**Figure S7** Low-temperature X-band ESR spectra of $Sr_{1-x}La_xCu_2(BO_3)_2$ single crystals with nominal (**a**) $x$ = 0.02, (**b**) 0.03, (**c**) 0.10, and (**d**) 0.15 at selected temperatures 5, 10 and 20 K, showing the development of the characteristic impurity signal correlated to intrinsic impurities−isolated dimer free $Cu^{2+}$ spins. ........ S12

**Figure S8** (**a**) The logarithmic temperature behavior of the X-band ESR linewidth of the main dimer-lattice signal. (**b**) The temperature behavior of $g$-factor for the interacting $Cu^{2+}$−$Cu^{2+}$ pair component for $Sr_{1-x}La_xCu_2(BO_3)_2$ single crystals with nominal $x$ = 0.02, 0.10, and 0.15. (**c**) The temperature behavior of X-band ESR linewidth for the isolated $Cu^{2+}$ impurity component, parallel ($\Delta B||$) and perpendicular ($\Delta B \perp$) to $c$ crystallographic axis for $Sr_{1-x}La_xCu_2(BO_3)_2$ single crystals with nominal $x$ = 0.02, 0.03, 0.10, and 0.15. (**d**) The temperature behavior of $g$-factor for the isolated $Cu^{2+}$ impurity component, parallel ($g||$) and perpendicular ($g \perp$) to $c$ crystallographic axis for $Sr_{1-x}La_xCu_2(BO_3)_2$ single crystals with nominal $x$ = 0.02, 0.03, 0.10, and 0.15. ........ S13

**Figure S9** (**a**) X-band ESR spectrum of $Sr_{1-x}La_xCu_2(BO_3)_2$ single crystals with nominal $x$ = 0.05 at 5 K. (**b**) The temperature behavior of X-band ESR linewidth for the isolated $Cu^{2+}$ impurity component, parallel ($\Delta B||$) and perpendicular ($\Delta B \perp$) to $c$ crystallographic axis for nominal $x$ = 0.05. (**c**) Temperature dependence of the signal intensity of the isolated $Cu^{2+}$ impurity component for nominal $x$ = 0.05. (**d**) The temperature behavior of X-band ESR linewidth for the interacting $Cu^{2+}$−$Cu^{2+}$ pair component for nominal $x$ = 0.05. ........ S14

**Table S1** Room-temperature structural parameters extracted from Rietveld refinement analyses of synchrotron PXRD data collected on crushed $Sr_{1-x}La_xCu_2(BO_3)_2$ single crystals of all studied nominal doping concentrations, $x = 0.02–0.15$, compared with polycrystalline undoped $SrCu_2(BO_3)_2$ according to ref. [1]. The refinements were performed in the $I\bar{4}2m$ space group.

| Nominal $x$ | $a$, $b$ (Å) | $c$ (Å) | $V$ (Å³) |
|---|---|---|---|
| 0 | 8.9932(1) | 6.6509(1) | 537.91(2) |
| 0.02 | 8.9932(1) | 6.6498(9) | 537.82(1) |
| 0.03 | 8.9929(1) | 6.6500(1) | 537.81(2) |
| 0.04 | 8.9928(5) | 6.6500(4) | 537.80(9) |
| 0.05 | 8.9922(1) | 6.6500(1) | 537.72(2) |
| 0.10 | 8.9878(1) | 6.6458(1) | 536.85(2) |
| 0.15 | 8.9868(1) | 6.6454(1) | 536.70(1) |

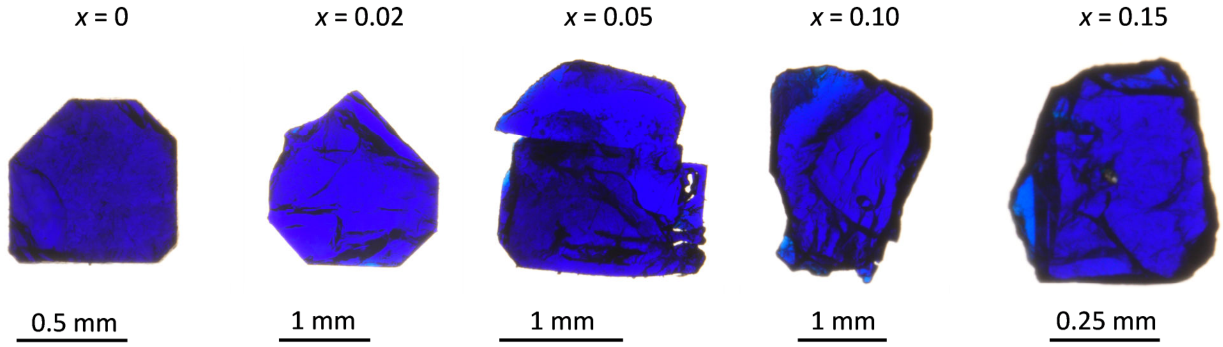


**Figure S1** Photographs of the blue La-doped $SrCu_2(BO_3)_2$ (in mol%) single crystals grown in this study.

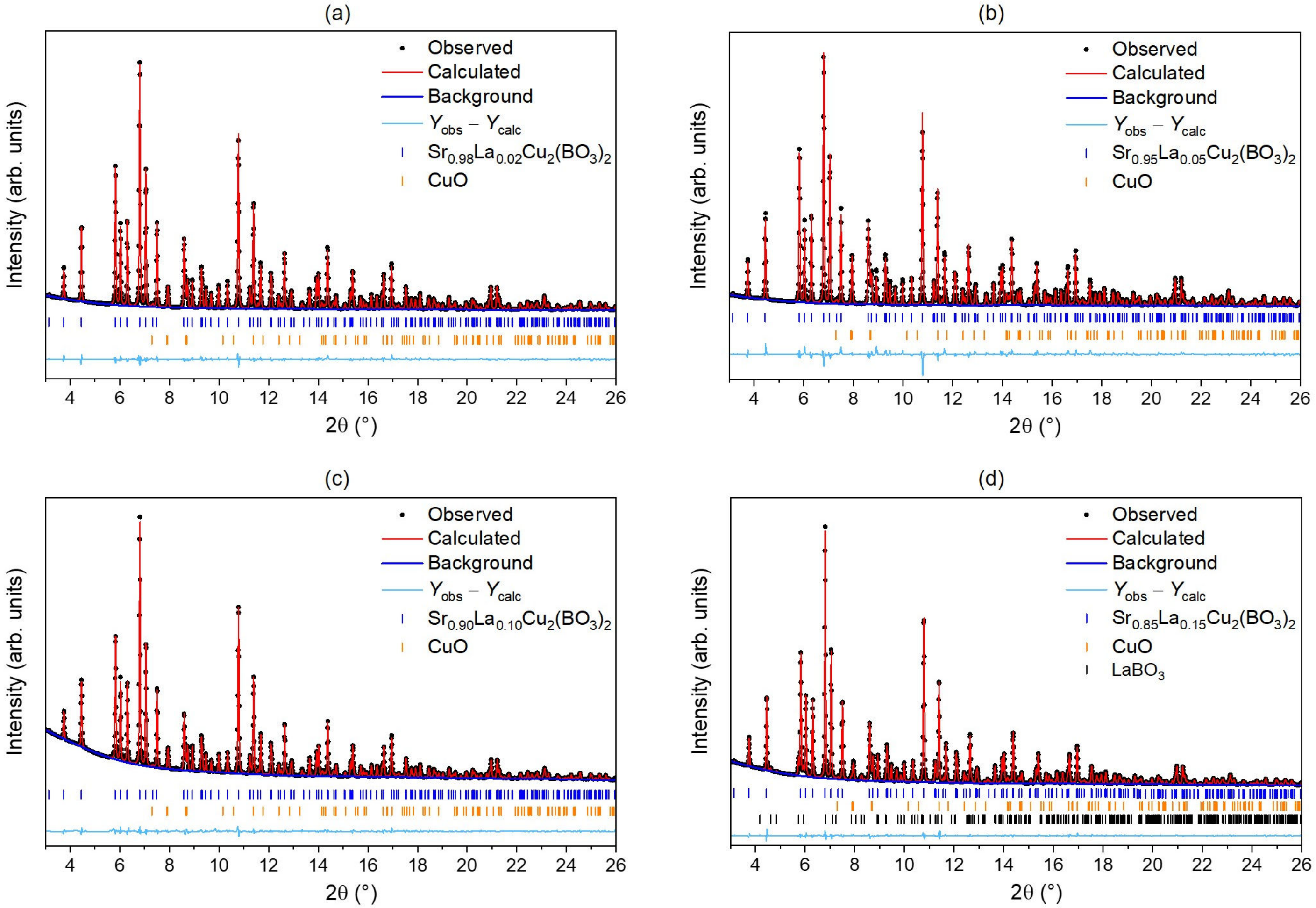


**Figure S2** Rietveld refinement profiles of synchrotron XRD data (λ = 0.3502 Å) collected on crushed $Sr_{1-x}La_xCu_2(BO_3)_2$ single crystals with selected nominal doping concentrations (**a**) $x$ = 0.02, (**b**) 0.05, (**c**) 0.10, and (**d**) 0.15, collected at room temperature. The refinements were performed using the $I\bar{4}2m$ space group.

**Table S2** Detailed results of Rietveld refinement analyses of synchrotron PXRD data collected on crushed $Sr_{1-x}La_xCu_2(BO_3)_2$ single crystals of selected nominal doping concentrations $x$ = 0.02, 0.05, 0.10 and 0.15. The data were refined using the $I\bar{4}2m$ space group.

| | $Sr_{0.98}La_{0.02}Cu_2(BO_3)_2$ | $Sr_{0.95}La_{0.05}Cu_2(BO_3)_2$ | $Sr_{0.90}La_{0.10}Cu_2(BO_3)_2$ | $Sr_{0.85}La_{0.15}Cu_2(BO_3)_2$ |
|---|---|---|---|---|
| ***a*, *b* (Å)** | 8.9932(1) | 8.9922(1) | 8.9878(1) | 8.9868(1) |
| ***c* (Å)** | 6.6498(1) | 6.6500(1) | 6.6458(1) | 6.6454(1) |
| ***V* (Å³)** | 537.82(1) | 537.72(2) | 536.85(2) | 536.70(1) |
| **$R_{wp}$ (%)** | 3.35 | 4.95 | 2.25 | 2.38 |
| **Atom** | x/y/z<br>Occ.<br>$U_{11}/U_{22}/U_{33}$ | x/y/z<br>Occ.<br>$U_{11}/U_{22}/U_{33}$ | x/y/z<br>Occ.<br>$U_{11}/U_{22}/U_{33}$ | x/y/z<br>Occ.<br>$U_{11}/U_{22}/U_{33}$ |
| **Sr** | 0/0.5/0<br>0.991*<br>0.0052(8)/0.0038(8)/0.0088(7) | 0/0.5/0<br>0.983*<br>0.0045(10)/0.0021(10)/0.0081(8) | 0/0.5/0<br>0.980*<br>0.0082(10)/0.0082(10)/0.0092(9) | 0/0.5/0<br>0.973*<br>0.0080(8)/0.0062(8)/0.0120(7) |
| **La** | 0/0.5/0<br>0.009*<br>0.0052(9)/0.0038(8)/0.0088(7) | 0/0.5/0<br>0.017*<br>0.0045(10)/0.0021(10)/0.0081(8)<br>0/0/0 | 0/0.5/0<br>0.020*<br>0.0082(11)/0.0082(10)/0.0092(9) | 0/0.5/0<br>0.027*<br>0.0085(8)/0.0062(8)/0.0120(7) |
| **Cu** | 0.11439(7)/0.11439(7)/ 0.27991(19)<br>1.00<br>0.0039(3)/0.0039/0.0162(8) | 0.11418(8)/0.11418/0.28199(20)<br>1.00<br>0.0008(4)/0.0008/0.0156(9) | 0.11439(8)/0.11439(8)/0.27927(26)<br>1.00<br>0.0046(4)/0.0046(4)/0.0269(12) | 0.11410(6)/0.11410(6)/0.27930(20)<br>1.00<br>0.0030(3)/0.0030(3)/0.0269(10) |
| **B** | 0.2966(5)/0.2966(6)/0.2467(16)<br>1.00<br>0.0021(25)/0.0021(15)/0.007(5) | 0.2928(6)/0.29278/0.2329(21)<br>1.00<br>0.0248(31)/0.0248/0.008(5) | 0.2938(7)/0.2938(7)/0.2547(26)<br>1.00<br>0.0164/0.0164/0.0050 | 0.2934(5)/0.2934(5)/0.2485(19)<br>1.00<br>0.0148/0.0148/0.0050 |
| **O1** | 0.4010(4)/0.4010(4)/0.2073(10)<br>1.00<br>0.0085(19)/0.0085/0.061(6) | 0.4029(4)/0.402913/0.2061(16)<br>1.00<br>0.0088(26)/0.0088/0.188(11) | 0.4013(4)/0.4013(4)/0.2111(14)<br>1.00<br>0.0020(21)/0.0020(21)/0.054(7) | 0.40166(29)/0.40166(29)/0.2136(11)<br>1.00<br>0.0032(15)/0.0032(15)/0.054(5) |
| **O2** | 0.32722(27)/0.14648(30)/0.2588(10)<br>1.00<br>0.0070(17)/0.0022(18)/0.0129(22) | 0.32785(4)/0.14367/0.26146<br>1.00<br>0.0058/0.0089/0.0169 | 0.32647(31)/0.1461(3)/0.2584(14)<br>1.00<br>0.0048(19)/0.0038(20)/0.0245(29) | 0.32701(26)/0.14574(28)/0.2588(10)<br>1.00<br>0.0032(14)/0.0023(15)/0.0259(24) |

*The fractional occupancies of Sr and La were fixed to the effective values, semi-quantitatively determined from SEM-EDS point analyses (Table S3).

**Table S3** The average semi-quantitative atomic % values of Sr, Cu, O and La in $Sr_{1-x}La_xCu_2(BO_3)_2$ single crystals with nominal $x$ = 0–0.15, as obtained from SEM-EDS point analyses, together with La/Cu, La/Sr and Cu/Sr ratios, versus the nominal values. The data was averaged from several crystals per concentration with approximatively 20 points collected on each crystal. Due to the low atomic number of boron, this element could not be detected by EDS and was therefore omitted from the semi-quantitative analysis.

| Element | Concentration (at%) | | | | | | | | | | | | | |
|---|---|---|---|---|---|---|---|---|---|---|---|---|---|---|
| | $x$ = 0 | | $x$ = 0.02 | | $x$ = 0.03 | | $x$ = 0.04 | | $x$ = 0.05 | | $x$ = 0.10 | | $x$ = 0.15 | |
| | Effective | Nominal | Effective | Nominal | Effective | Nominal | Effective | Nominal | Effective | Nominal | Effective | Nominal | Effective | Nominal |
| **Sr** | 13.9(4) | 11.11 | 12.6(5) | 10.89 | 13.1(7) | 10.78 | 13.1(3) | 10.67 | 12.4(5) | 10.56 | 12.4(2) | 10.00 | 12.4(2) | 9.44 |
| **Cu** | 28(2) | 22.22 | 27(1) | 22.22 | 26(3) | 22.22 | 25(1) | 22.22 | 27(2) | 22.22 | 26.0(9) | 22.22 | 26(1) | 22.22 |
| **O** | 59(2) | 66.67 | 61(2) | 66.67 | 61(3) | 66.67 | 62(1) | 66.67 | 60(2) | 66.67 | 62(1) | 66.67 | 62(1) | 66.67 |
| **La** | 0 | 0 | 0.10(2) | 0.22 | 0.16(5) | 0.33 | 0.15(3) | 0.44 | 0.18(6) | 0.56 | 0.22(3) | 1.11 | 0.30(6) | 1.67 |
| **Sum, %** | 100.9 | 100 | 100.7 | 100 | 100.26 | 100 | 100.25 | 100 | 99.58 | 100 | 100.62 | 100 | 100.7 | 100 |
| **La/Cu** | 0 | 0 | 0.0036(8) | 0.01 | 0.006(2) | 0.015 | 0.006(1) | 0.02 | 0.007(2) | 0.025 | 0.009(1) | 0.05 | 0.012(2) | 0.075 |
| **La/Sr** | 0 | 0 | 0.008(2) | 0.02 | 0.012(3) | 0.03 | 0.011(2) | 0.04 | 0.015(4) | 0.05 | 0.018(2) | 0.11 | 0.025(5) | 0.18 |
| **Cu/Sr** | 1.99(9) | 2 | 2.10(7) | 2.04 | 2.0(1) | 2.06 | 1.91(4) | 2.08 | 2.2(1) | 2.10 | 2.06(5) | 2.22 | 2.08(6) | 2.35 |
| **La mol%** | 0 | 0 | 0.9(2) | 2 | 1.4(5) | 3 | 1.3(2) | 4 | 1.7(5) | 5 | 2.0(3) | 10 | 2.7(6) | 15 |

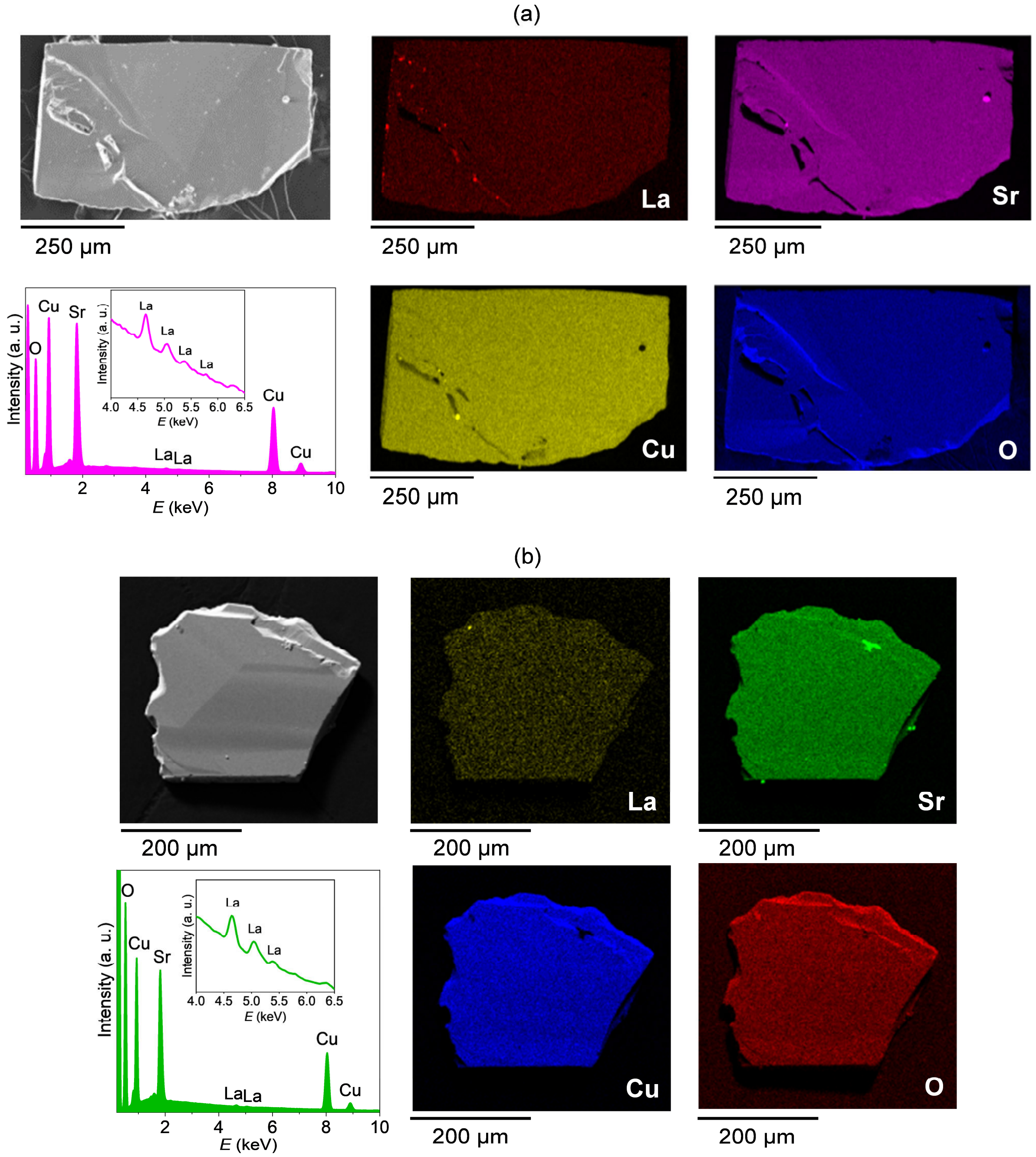


**Figure S3** An SEM image and EDS elemental mapping analysis of a $Sr_{1-x}La_xCu_2(BO_3)_2$ single crystal with nominal $x = 0.04$ (**a**) and 0.10 (**b**) reveal a relatively homogeneous distribution of La within the $SrCu_2(BO_3)_2$ matrix. Few $LaBO_3$ impurity particles are observed on the surface. The corresponding EDS sum spectra clearly show the presence of La emission lines for both a) and b) samples. The insets show a magnified region where the La emission lines are distinctly visible. Few CuO impurity particles are additionally visible in a).

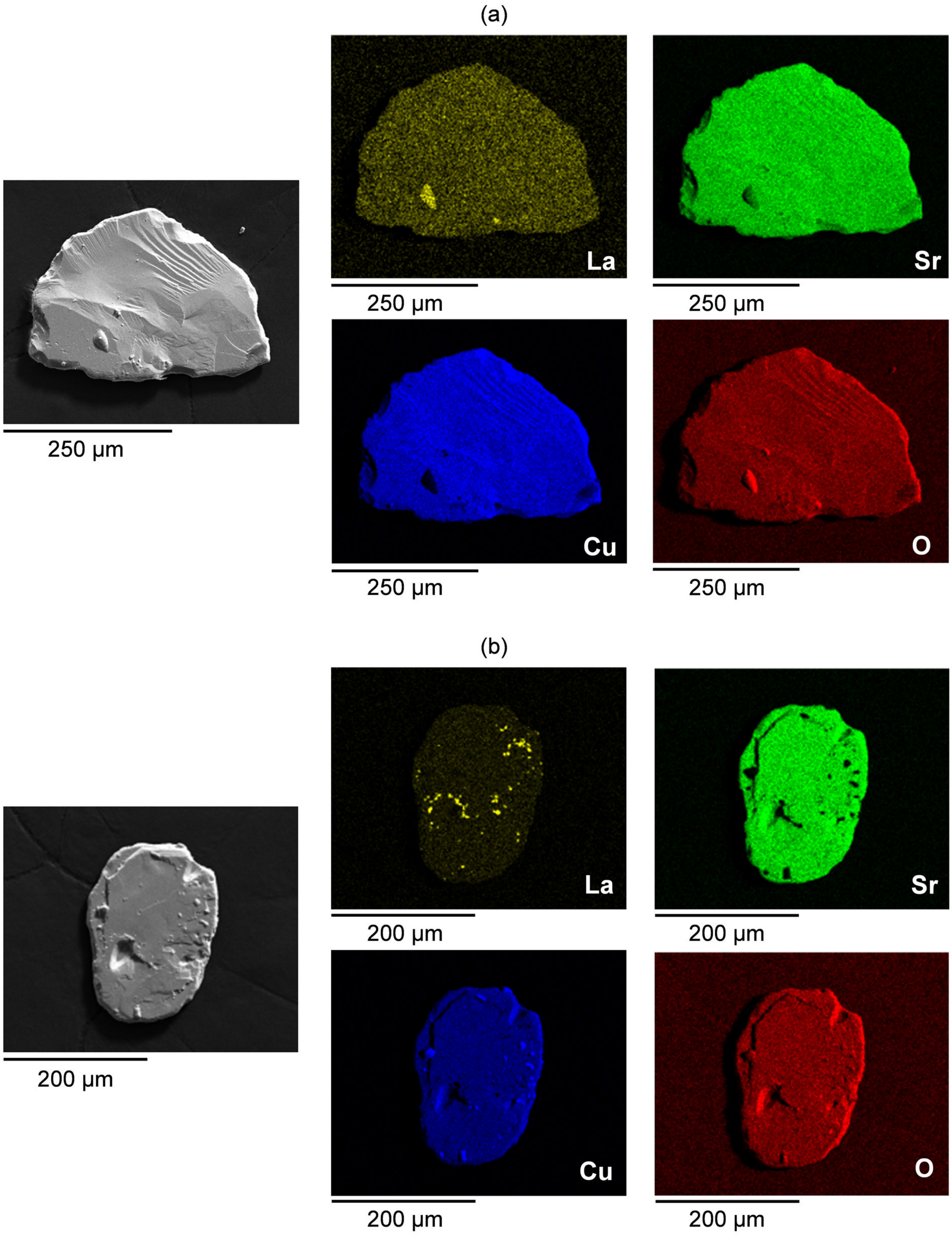


**Figure S4** An SEM image (left) and EDS elemental mapping analysis (right) of a $Sr_{1-x}La_xCu_2(BO_3)_2$ single crystal with nominal $x$ = 0.15, showing: (**a**) the presence of $LaBO_3$ impurity particles on the crystal surface and (**b**) the presence of both CuO and $LaBO_3$ impurity particles on the crystal surface.

**Table S4** Results of the low-temperature magnetic susceptibility data fits (2–6 K range) for $Sr_{1-x}La_xCu_2(BO_3)_2$ single crystals with nominal $x$ = 0–0.15 (Equation 3 in the main text). Five fixed θ' values were used due to a high correlation with C'.

| Nominal $x$ | C' (emu K/mol) | $\chi_0$ (emu/mol) | θ' | $\Delta$ (K) | $a$ (emu/mol) |
|---|---|---|---|---|---|
| $x$ = 0 | $3.4(1) \cdot 10^{-4}$ | $7.1(6) \cdot 10^{-5}$ | 0 | 28.7(3) | $8.3(4) \cdot 10^{-2}$ |
| | $4.1(2) \cdot 10^{-4}$ | $5.7(6) \cdot 10^{-5}$ | −0.25 | 28.5(3) | $8.1(4) \cdot 10^{-2}$ |
| | $4.9(2) \cdot 10^{-4}$ | $4.4(6) \cdot 10^{-5}$ | −0.5 | 28.3(3) | $7.9(4) \cdot 10^{-2}$ |
| | $5.7(2) \cdot 10^{-4}$ | $3.1(7) \cdot 10^{-5}$ | −0.75 | 28.2(3) | $7.8(3) \cdot 10^{-2}$ |
| | $6.6(3) \cdot 10^{-4}$ | $1.8(7) \cdot 10^{-5}$ | −1 | 28.1(3) | $7.7(3) \cdot 10^{-2}$ |
| $x$ = 0.02 | $1.95(1) \cdot 10^{-3}$ | $3.09(6) \cdot 10^{-4}$ | 0 | 26.7(3) | $6.6(3) \cdot 10^{-2}$ |
| | $2.37(1) \cdot 10^{-3}$ | $2.31(5) \cdot 10^{-4}$ | −0.25 | 25.9(2) | $5.9(2) \cdot 10^{-2}$ |
| | $2.82(2) \cdot 10^{-3}$ | $1.52(6) \cdot 10^{-4}$ | −0.5 | 25.2(2) | $5.4(2) \cdot 10^{-2}$ |
| | $3.32(2) \cdot 10^{-3}$ | $7.2(7) \cdot 10^{-5}$ | −0.75 | 24.7(2) | $5.1(2) \cdot 10^{-2}$ |
| | $3.86(3) \cdot 10^{-3}$ | $-9(9) \cdot 10^{-6}$ | −1 | 24.2(2) | $4.8(2) \cdot 10^{-2}$ |
| $x$ = 0.03 | $2.46(2) \cdot 10^{-3}$ | $3.83(8) \cdot 10^{-4}$ | 0 | 25.3(3) | $5.4(2) \cdot 10^{-2}$ |
| | $2.99(2) \cdot 10^{-3}$ | $2.82(6) \cdot 10^{-4}$ | −0.25 | 24.4(2) | $4.8(1) \cdot 10^{-2}$ |
| | $3.58(2) \cdot 10^{-3}$ | $1.79(6) \cdot 10^{-4}$ | −0.5 | 23.6(2) | $4.4(1) \cdot 10^{-2}$ |
| | $4.22(2) \cdot 10^{-3}$ | $7.4(7) \cdot 10^{-5}$ | −0.75 | 23.0(2) | $4.1(1) \cdot 10^{-2}$ |
| | $4.91(3) \cdot 10^{-3}$ | $-3.2(9) \cdot 10^{-5}$ | −1 | 22.4(2) | $3.8(1) \cdot 10^{-2}$ |
| $x$ = 0.04 | $2.25(2) \cdot 10^{-3}$ | $3.65(9) \cdot 10^{-4}$ | 0 | 25.4(4) | $5.0(3) \cdot 10^{-2}$ |
| | $2.74(3) \cdot 10^{-3}$ | $2.73(9) \cdot 10^{-4}$ | −0.25 | 24.5(3) | $4.5(2) \cdot 10^{-2}$ |
| | $3.27(3) \cdot 10^{-3}$ | $1.8(1) \cdot 10^{-4}$ | −0.5 | 23.7(3) | $4.1(2) \cdot 10^{-2}$ |
| | $3.86(4) \cdot 10^{-3}$ | $8(1) \cdot 10^{-5}$ | −0.75 | 23.1(3) | $3.8(2) \cdot 10^{-2}$ |
| | $4.49(5) \cdot 10^{-3}$ | $-1(1) \cdot 10^{-5}$ | −1 | 22.5(3) | $3.6(2) \cdot 10^{-2}$ |
| $x$ = 0.05 | $2.39(2) \cdot 10^{-3}$ | $4.12(6) \cdot 10^{-4}$ | 0 | 24.8(3) | $4.4(2) \cdot 10^{-2}$ |
| | $2.90(1) \cdot 10^{-3}$ | $3.17(5) \cdot 10^{-4}$ | −0.25 | 23.6(2) | $3.7(1) \cdot 10^{-2}$ |
| | $3.46(2) \cdot 10^{-3}$ | $2.16(5) \cdot 10^{-4}$ | −0.5 | 22.8(2) | $3.37(8) \cdot 10^{-2}$ |
| | $4.09(2) \cdot 10^{-3}$ | $1.13(7) \cdot 10^{-4}$ | −0.75 | 22.1(2) | $3.12(8) \cdot 10^{-2}$ |
| | $4.68(3) \cdot 10^{-3}$ | $3.4(8) \cdot 10^{-5}$ | −1 | 22.0(2) | $3.15(9) \cdot 10^{-2}$ |
| $x$ = 0.10 | $2.50(2) \cdot 10^{-3}$ | $5.66(7) \cdot 10^{-4}$ | 0 | 23.8(3) | $3.0(1) \cdot 10^{-2}$ |
| | $3.04(1) \cdot 10^{-3}$ | $4.60(5) \cdot 10^{-4}$ | −0.25 | 22.6(2) | $2.60(7) \cdot 10^{-2}$ |
| | $3.64(1) \cdot 10^{-3}$ | $3.51(4) \cdot 10^{-4}$ | −0.5 | 21.6(1) | $2.33(4) \cdot 10^{-2}$ |
| | $4.31(2) \cdot 10^{-3}$ | $2.39(5) \cdot 10^{-4}$ | −0.75 | 20.8(1) | $2.14(4) \cdot 10^{-2}$ |
| | $5.04(2) \cdot 10^{-3}$ | $1.26(7) \cdot 10^{-5}$ | −1 | 20.1(2) | $2.00(5) \cdot 10^{-2}$ |
| $x$ = 0.15 | $2.51(2) \cdot 10^{-3}$ | $7.3(1) \cdot 10^{-4}$ | 0 | 22.8(4) | $2.8(2) \cdot 10^{-2}$ |
| | $3.05(3) \cdot 10^{-3}$ | $6.3(1) \cdot 10^{-4}$ | −0.25 | 21.8(4) | $2.5(1) \cdot 10^{-2}$ |
| | $3.64(4) \cdot 10^{-3}$ | $5.2(1) \cdot 10^{-4}$ | −0.5 | 21.0(4) | $2.3(1) \cdot 10^{-2}$ |
| | $4.30(5) \cdot 10^{-3}$ | $4.1(2) \cdot 10^{-4}$ | −0.75 | 20.3(4) | $2.2(1) \cdot 10^{-2}$ |
| | $5.02(6) \cdot 10^{-3}$ | $3.0(2) \cdot 10^{-4}$ | −1 | 19.7(4) | $2.0(1) \cdot 10^{-2}$ |

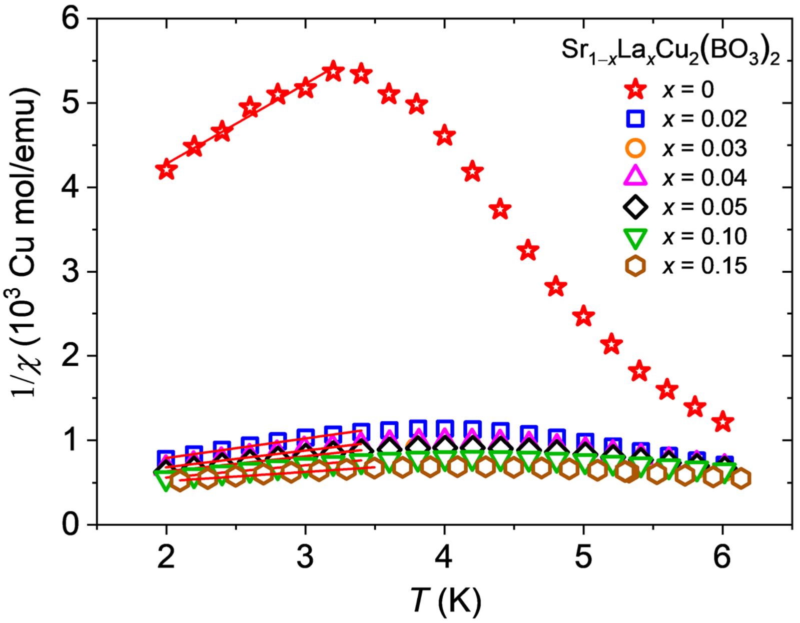


**Figure S5** Inverse magnetic susceptibility data for $Sr_{1-x}La_xCu_2(BO_3)_2$ single crystals with nominal $x$ = 0–0.15, displaying low-temperature Curie–Weiss fits performed in the 2–3.4 K interval to estimate intrinsic $Cu^{2+}$ ($S$ = ½) impurities.

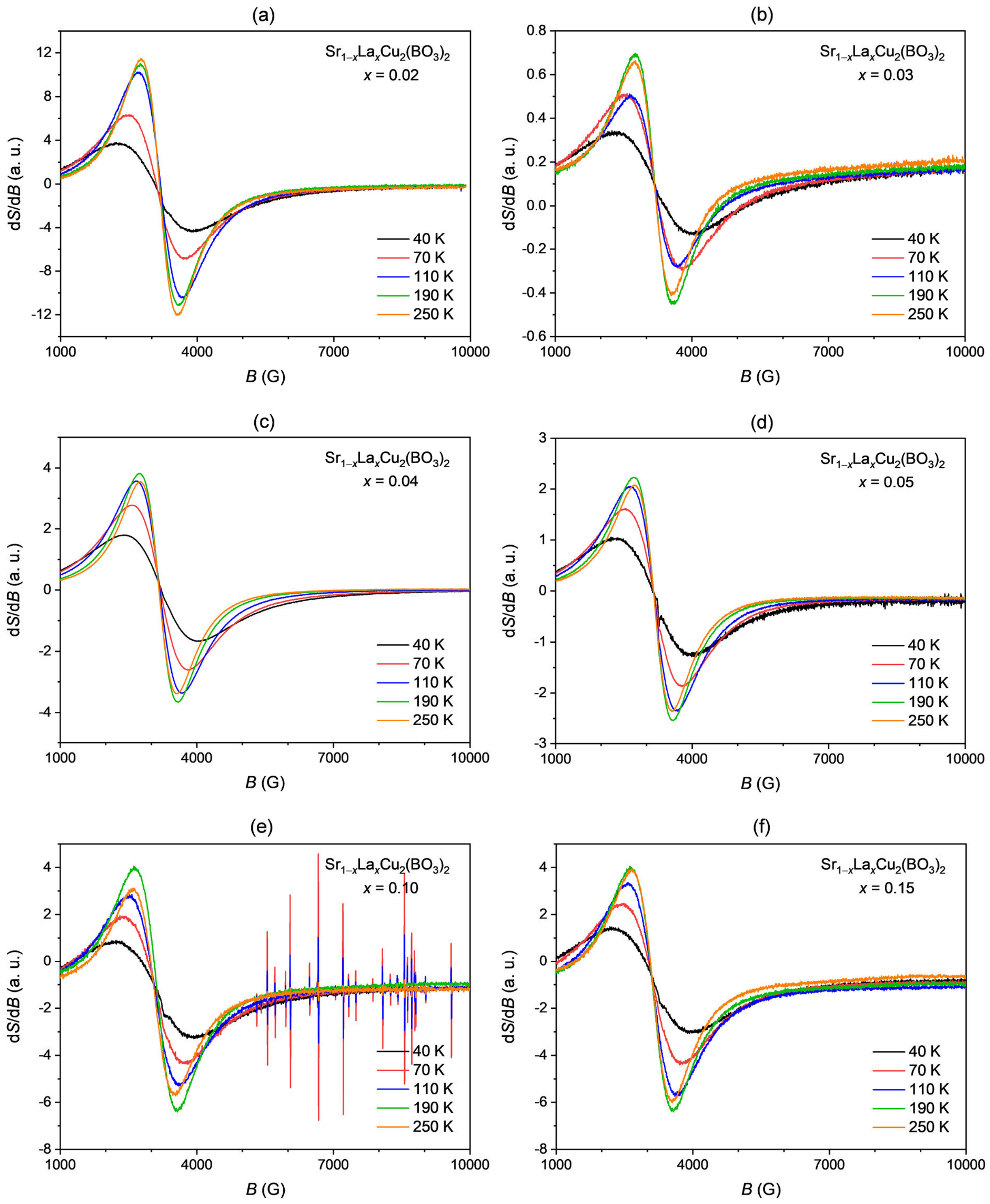


**Figure S6** X-band ESR spectra of $Sr_{1-x}La_xCu_2(BO_3)_2$ single crystals with nominal (**a**) $x$ = 0.02, (**b**) 0.03, (**c**) 0.04, (**d**) 0.05, (**e**) 0.10 and (**f**) 0.15, measured as quasi-polycrystalline samples at temperatures 40, 70, 110, 170 and 250 K, which show the dominant signal of the dimer lattice at high temperatures. Additional narrow absorption lines for $x$ = 0.10 sample indicate the presence of an extrinsic paramagnetic impurity, not observed in PXRD or EDS-SEM.

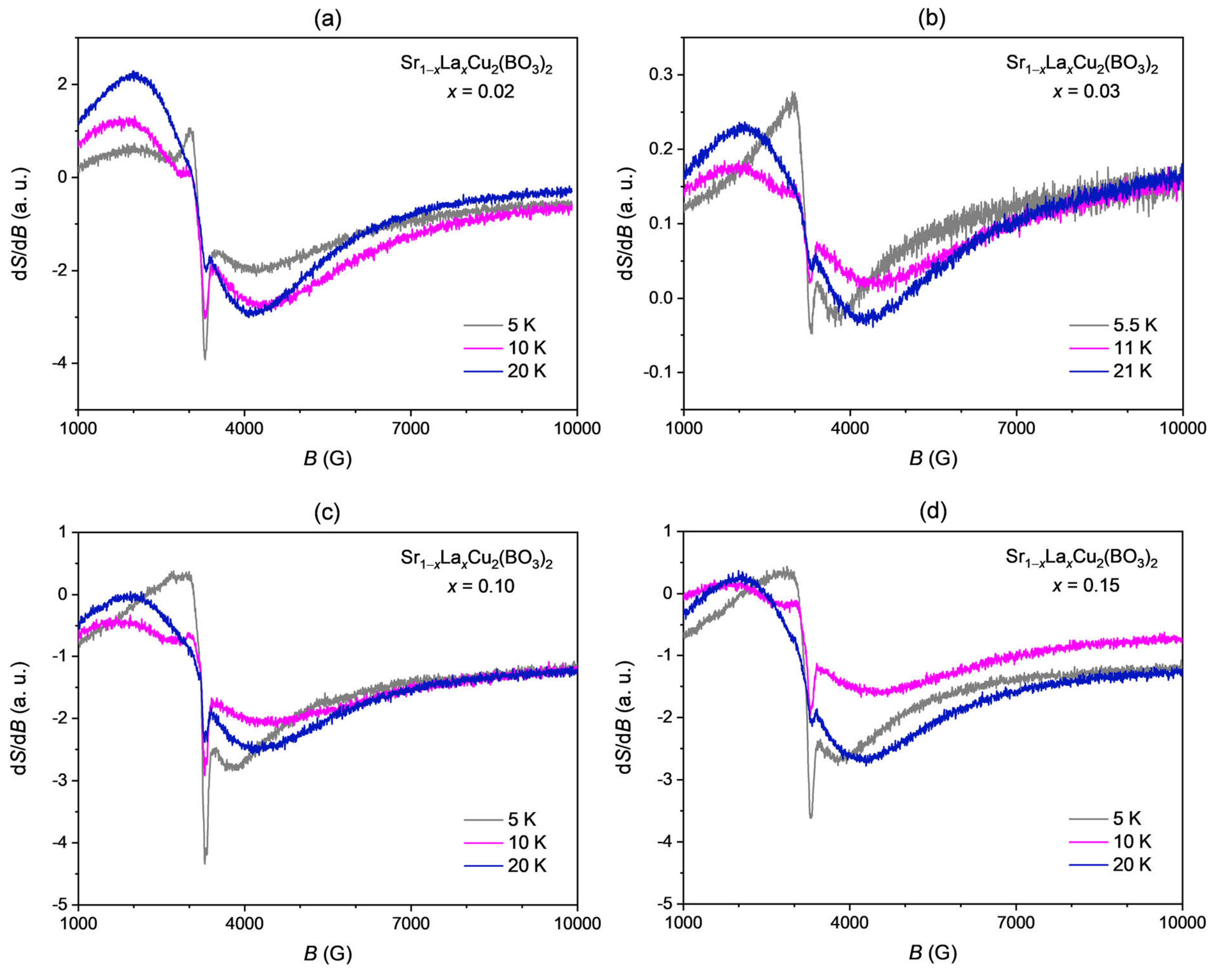


**Figure S7** Low-temperature X-band ESR spectra of $Sr_{1-x}La_xCu_2(BO_3)_2$ single crystals with nominal (**a**) $x = 0.02$, (**b**) 0.03, (**c**) 0.10, and (**d**) 0.15 at selected temperatures 5, 10 and 20 K, showing the development of the characteristic impurity signal correlated to intrinsic impurities−isolated dimer free $Cu^{2+}$ spins. Comparing the spectra at 5 and 10 K, an additional broadening is observed at 5 K, indicating the presence of an extra component associated with interactions between dimer-free $Cu^{2+}$–$Cu^{2+}$ pairs.

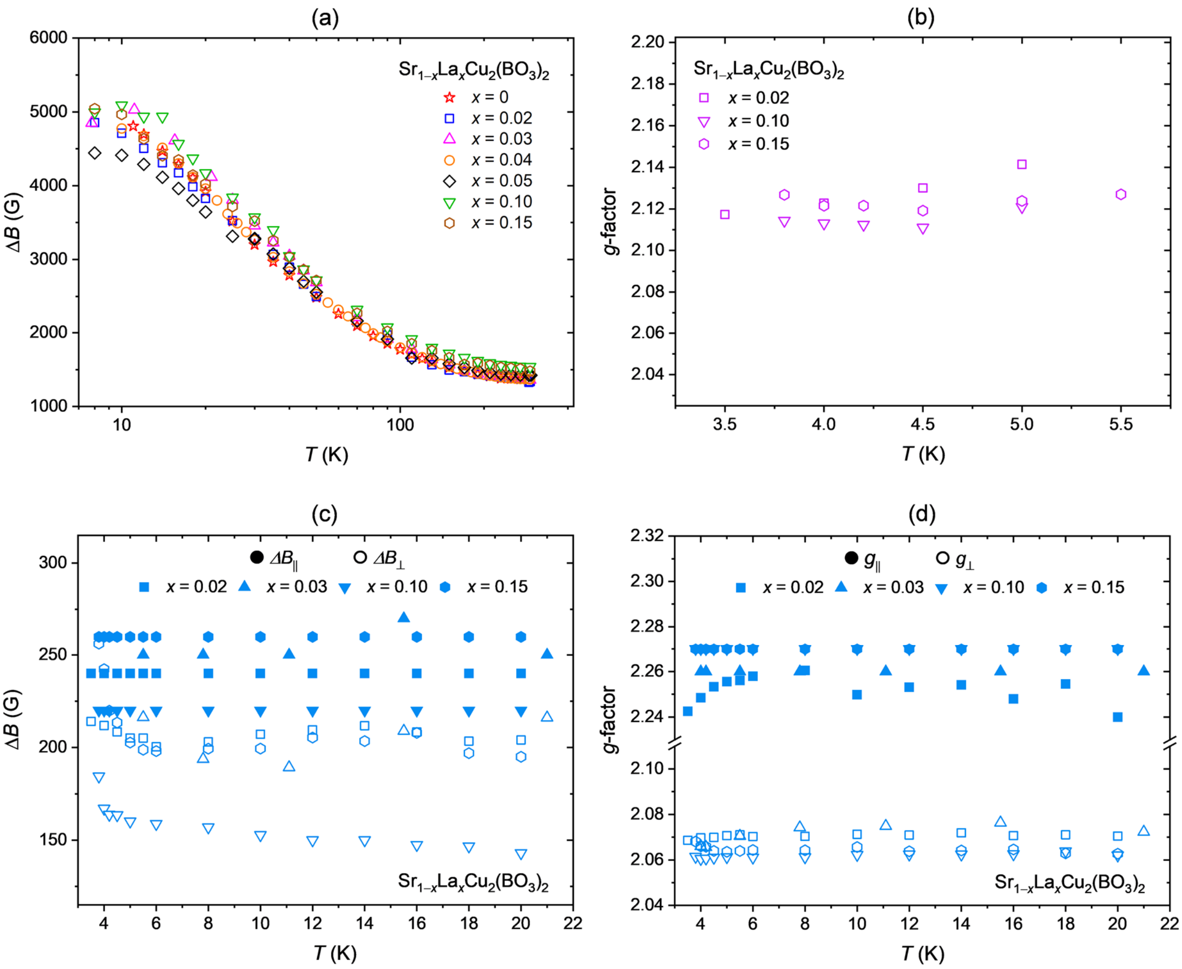


**Figure S8** (**a**) The logarithmic temperature behavior of the X-band ESR linewidth of the main dimer-lattice signal. (**b**) The temperature behavior of *g*-factor for the interacting $Cu^{2+}$–$Cu^{2+}$ pair component for $Sr_{1-x}La_xCu_2(BO_3)_2$ single crystals with nominal $x = 0.02$, 0.10, and 0.15. (**c**) The temperature behavior of X-band ESR linewidth for the isolated $Cu^{2+}$ impurity component, parallel ($\Delta B_{||}$) and perpendicular ($\Delta B_{\perp}$) to *c* crystallographic axis for $Sr_{1-x}La_xCu_2(BO_3)_2$ single crystals with nominal $x = 0.02$, 0.03, 0.10, and 0.15. (**d**) The temperature behavior of *g*-factor for the isolated $Cu^{2+}$ impurity component, parallel ($g_{||}$) and perpendicular ($g_{\perp}$) to *c* crystallographic axis for $Sr_{1-x}La_xCu_2(BO_3)_2$ single crystals with nominal $x = 0.02$, 0.03, 0.10, and 0.15.

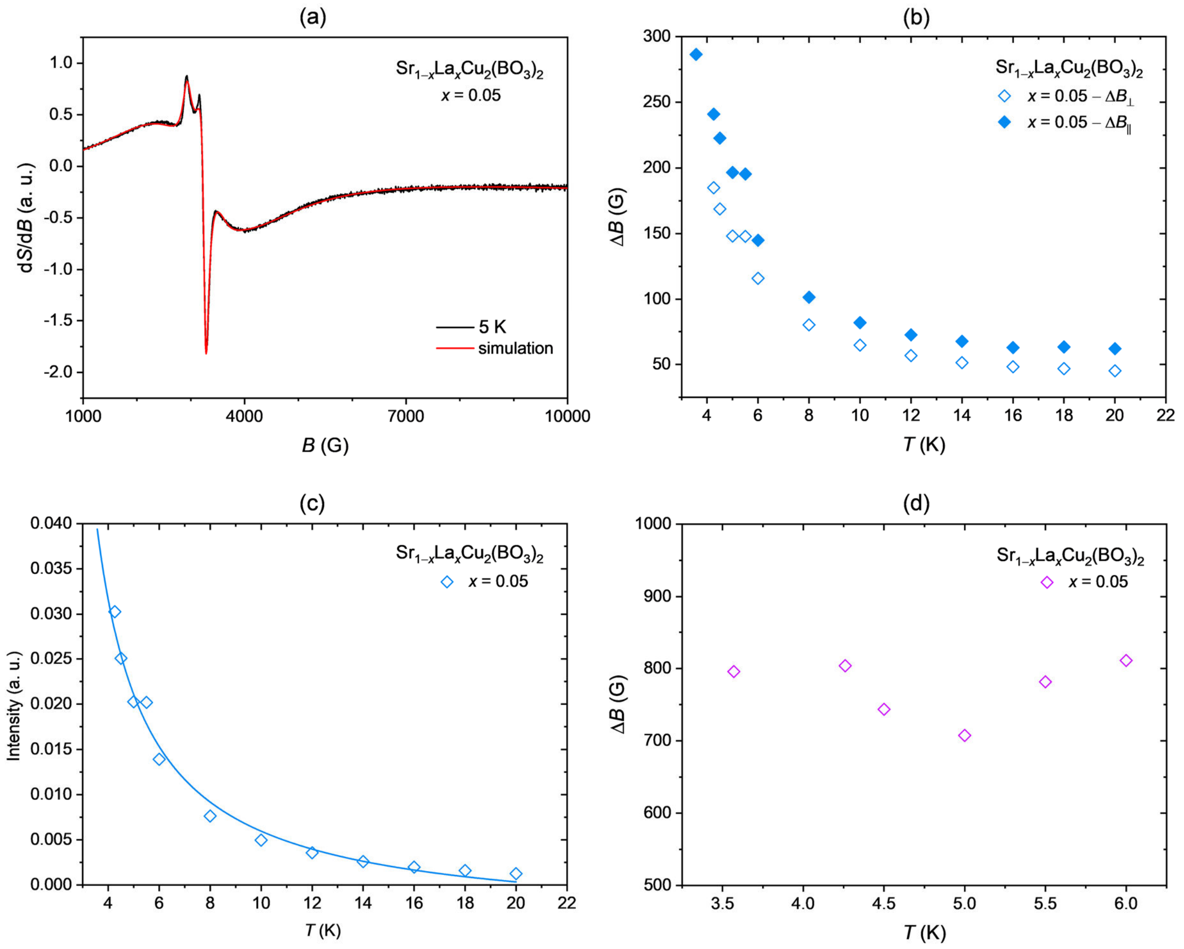


**Figure S9** (**a**) X-band ESR spectrum of $Sr_{1-x}La_xCu_2(BO_3)_2$ single crystals with nominal $x = 0.05$ at 5 K, showing a clear uniaxial anisotropy opposed to samples with other nominal doping concentrations, and its simulation as a sum of three components. (**b**) The temperature behavior of X-band ESR linewidth for the isolated $Cu^{2+}$ impurity component, parallel ($\Delta B_{||}$) and perpendicular ($\Delta B_{\perp}$) to *c* crystallographic axis for nominal $x = 0.05$, showing a significant increase in both crystallographic directions with the decreasing temperature, contrary to the rest of the samples. (**c**) Temperature dependence of the signal intensity of the isolated $Cu^{2+}$ impurity component for nominal $x = 0.05$, which follows a Curie–Weiss law, giving a low Curie-Weiss temperature. This indicates minimal spin correlations, aligning with the rest of the samples. (**d**) The temperature behavior of X-band ESR linewidth for the interacting $Cu^{2+}$–$Cu^{2+}$ pair component for nominal $x = 0.05$. Contrary to the rest of the samples, the linewidth remains constant and does not show a decrease with the decreasing temperature.

## REFERENCES

[1] Šibav, L.; Gosar, Ž.; Knaflič, T.; Jagličić, Z.; King, G.; Nojiri, H.; Arčon, D.; Dragomir, M. Higher-Magnesium-Doping Effects on the Singlet Ground State of the Shastry-Sutherland $SrCu_2(BO_3)_2$. *Inorg. Chem.* **2024**, 63, 20335–20346. DOI: 10.1021/acs.inorgchem.4c02398
